# PCB: A pseudotemporal causality-based Bayesian approach to identify EMT-associated regulatory relationships of AS events and RBPs during breast cancer progression

**DOI:** 10.1371/journal.pcbi.1010939

**Published:** 2023-03-17

**Authors:** Liangjie Sun, Yushan Qiu, Wai-Ki Ching, Pu Zhao, Quan Zou

**Affiliations:** 1 Department of Mathematics, The University of Hong Kong, Hong Kong, China; 2 College of Mathematics and Statistics, Shenzhen University, Shenzhen, China; 3 College of Life and Health Sciences, Northeastern University, Shenyang, China; 4 Institute of Fundamental and Frontier Sciences, University of Electronic Science and Technology of China, Chengdu, China; Florida International University, UNITED STATES

## Abstract

During breast cancer metastasis, the developmental process epithelial-mesenchymal (EM) transition is abnormally activated. Transcriptional regulatory networks controlling EM transition are well-studied; however, alternative RNA splicing also plays a critical regulatory role during this process. Alternative splicing was proved to control the EM transition process, and RNA-binding proteins were determined to regulate alternative splicing. A comprehensive understanding of alternative splicing and the RNA-binding proteins that regulate it during EM transition and their dynamic impact on breast cancer remains largely unknown. To accurately study the dynamic regulatory relationships, time-series data of the EM transition process are essential. However, only cross-sectional data of epithelial and mesenchymal specimens are available. Therefore, we developed a pseudotemporal causality-based Bayesian (PCB) approach to infer the dynamic regulatory relationships between alternative splicing events and RNA-binding proteins. Our study sheds light on facilitating the regulatory network-based approach to identify key RNA-binding proteins or target alternative splicing events for the diagnosis or treatment of cancers. The data and code for PCB are available at: http://hkumath.hku.hk/~wkc/PCB(data+code).zip.

## Author summary

In this paper, pseudotime causality-based Bayesian (PCB) model is proposed to detect the regulatory relationships between alternative splicing events and RNA-binding proteins in the EM transition process of breast cancer. It is the first time to reveal the global role of alternative splicing events and RNA-binding proteins during EM transition, in which RNA-binding proteins can dynamically regulate alternative splicing. Furthermore, to address the challenges that lack of temporal information in sample-based transcriptomic data, we propose to decode the latent temporal information underlying cancer progression through ordering patient sample based on transcriptomic similarity, and design a latent-temporal progression-based Bayesian method to infer alternative splicing regulatory network from sample based transcriptomic data of cancer patients. We inferred dynamic regulatory relationships between EM transition-associated alternative splicing events and RNA-binding proteins. Overall, we determined that ZCCHC24, PIWIL4, and TDRD10 may be key RNA-binding proteins in the EM transition process of breast cancer. In addition, we studied the dynamic regulatory relationships between alternative splicing events of the CD44 gene and RNA-binding proteins (hnRNPM, ESRP1 and ESRP2). Our study suggests that inferring dynamic progression trajectory from static expression data of tumor samples helps to uncover regulatory mechanism underlying cancer progression. In addition, our study proposes a new and effective way to characterize cancer progression by clinical transcriptomic data modeling and facilitate the incorporation of a regulatory network-based approach into precision medicine.

## Introduction

Tumor metastasis, which involves the spread of cancer cells from the original site to other organs, has always been the major cause of death in cancer patients. During tumor metastasis, tumor cells hijack the development of epithelial-mesenchymal (EM) transition to attack surrounding tissues and migrate to distant organs [1, 2]. In recent years, increasing evidences have shown that EM transition is abnormally activated in cancer cells [1, 2]. Furthermore, it is generally believed that EM transition may contribute to breast cancer metastasis. EM transition is a cellular process in which epithelial cells obtain mesenchymal phenotype and behavior after epithelial characteristics are down-regulated. EM transition often appears in biological processes such as tissue repair and wound healing. EM transition is regulated by four major regulatory systems: alternative splicing, transcriptional control, post-translational control, and noncoding RNA [3]. Here, the transcriptional regulatory network controlling EM transition has been well studied [2, 4, 5]. However, alternative RNA splicing also plays a critical regulatory role during this process. A comprehensive understanding of alternative splicing events and the RNA-binding proteins that dynamically regulate it during EM transition and their impact on breast cancer remains largely unknown.

Alternative splicing, also referred to as selective RNA splicing or differential splicing, is the process of choosing diverse splicing site combinations in a precursor messenger RNA (pre-mRNA) to produce variably spliced mRNAs. Proteins with different sequences and activities are encoded through these mRNAs, but all these mRNAs come from a single gene. In other words, alternative splicing is an important biological process by which cells can express several variants, also known as isoforms, of a single gene. Each splicing variant gives rise to a different protein with a unique structure that can perform different functions and respond to internal and environmental needs. Alternative splicing is both a vital mechanism for development and cell type-specific control of gene expression, and a mechanism for enriching proteome diversity. Numerous studies have confirmed that alternative splicing changes dynamically during EM transition, and alternative splicing regulation is crucial in EM transition [6–10]. It was reported that almost all human genes undergo alternative splicing, and alternatively spliced isomers play different functional roles in cells [11, 12].

Cis-elements located near the pre-mRNAs variable exons regulate alternative splicing. These various cis-elements are identified by cognate RNA-binding proteins, which affect the recognition of splice sites by spliceosomes. Splicing-regulatory RNA-binding protein has a positive or negative impact on the inclusion of alternative exons. RNA-binding proteins often interact in complexes to regulate splicing regulation [13–15]. Hence, the relationships between RNA-binding proteins and their target alternative splicing events are largely context-dependent, and cracking the “splicing code” is an urgent problem in this field [16]. It is necessary to develop a computational method to get how and to what extent diverse groups of RNA-binding proteins regulate given alternative splicing events.

Recently some effective models [17, 18] have been proposed to infer the relationships between alternative splicing events and RNA-binding proteins. However, they fail to reveal the dynamic relationships between alternative splicing events and RNA-binding proteins during the EM transition process, which is of great importance. To explore the dynamic regulatory relationships, people usually apply time-course expression data. On the other hand, the lack of sufficient temporal information is prevalent in most of the available omics data from cross-sectional studies of cancer patients, which is the main reason why it is difficult to infer the association between alternative splicing events and RNA-binding proteins.

In order to infer gene dynamics and thus infer the sequence of cellular programs, collective process dynamics can be reconstructed by reordering cells according to some measure of expression similarity, which is known as pseudotemporal ordering. For example, in [19], Wanderlust was proposed to identify cellular trajectories, which can capture nonlinear behavior. It directly constructs a collection of *k*-nearest neighbor graphs in high-dimensional space, and then obtains the shortest path through this collection. However, this method has certain limitations. First, Wanderlust is designed to process the data from the expression of protein markers. In this case, the number of markers is relatively small (dozens, not hundreds), and markers are selected manually according to their prior knowledge of the process involved. Secondly, when constructing cellular trajectories, Wanderlust always assumes that these processes are non-branching. Moreover, in [20], latent-temporal progression-based Bayesian (PROB) method was proposed to infer gene regulatory network, which only uses gene expression and pathological information (stage information) to infer latent-temporal progression. However, this method only considers a single progression trajectory, which is insufficient in inferring the multi branch trajectory of disease progression or cell differentiation. In addition, this method cannot handle missing data in clinical transcriptome data.

Motivated by the above problems and challenges, in this study, based on the concept of pseudotime [19] and latent temporal [20], we propose a pseudotime causality-based Bayesian (PCB) model to identify the EM transition-associated alternative splicing events and RNA-binding proteins regulatory relationships, using cross-sectional data of epithelial and mesenchymal specimens in breast cancer. Apply this model to real dataset from [21]. Using this dataset, we first found EM transition-associated alternative splicing events and RNA-binding proteins in breast cancer, and studied the dynamic regulatory relationships between these alternative splicing events and RNA-binding proteins; then we revealed the dynamic regulatory relationships between the alternative splicing events of the CD44 gene and RNA-binding proteins (heterogeneous nuclear ribonucleoprotein M, hnRNPM; epithelial splicing regulatory protein 1, ESRP1; and epithelial splicing regulatory protein 2, ESRP2) during the EM transition process.

The main contribution can be summarized as follows: 1) this paper integrates alternative splicing events and RNA-binding proteins into a dynamical system for the first time, which more clearly reveals the regulatory relationships between alternative splicing events and RNA-binding proteins; 2) our study suggests a novel and effective way to identify key regulatory relationships of alternative splicing events and RNA-binding proteins of cancer progression based on cross-sectional data; 3) our study also proposes a novel strategy to identify target alternative splicing events or key RNA-binding proteins for the diagnosis or treatment of cancers; 4) pseudotime analysis is first applied to cross-sectional alternative splicing and RNA-binding protein expression data of epithelial and mesenchymal specimens. Although our pseudotime analysis is mainly based on the PROB model in [20], cross-sectional data with missing values is processed, and alternative splicing event and RNA-binding protein expression data are mapped into the same pseudotime trajectory.

## Methods

### Problem definition and method outline

**Fig 1:**
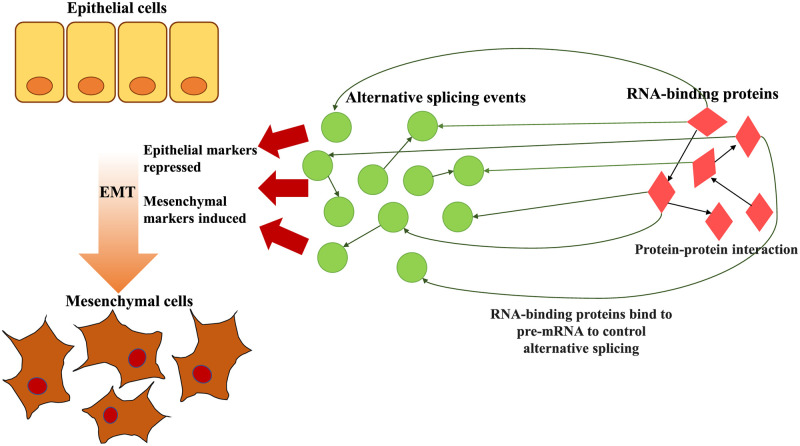
Pictorial representation of the EM transition process by incorporating alternative splicing events and RNA-binding proteins.

Based on the premise that alternative splicing plays an important regulatory role in the EM transition process and that RNA-binding proteins bind to pre-mRNA to regulate alternative splicing, it is necessary to propose a method that identifies the dynamic regulatory relationships between alternative splicing events and RNA-binding proteins in the EM transition process (Fig 1). To study the regulatory relationships, it is ideal to experimentally obtain EM transition time-series data. Unfortunately, collecting time-series experimental data is expensive. Currently, only cross-sectional data of epithelial and mesenchymal specimens are available.

Inspired by the above discussion, we proposed a PCB model in this paper. Based on this model, we studied the following two issues. First, we found EM transition-associated alternative splicing events and RNA-binding proteins in breast cancer and studied the dynamic regulatory relationships between these alternative splicing events and RNA-binding proteins. Second, the dynamic regulatory relationships between alternative splicing events of the CD44 gene and the RNA-binding proteins (hnRNPM, ESRP1 and ESRP2) during the EM transition process was revealed.

PCB consists of two major components. First, the time-series data requirement is solved by performing pseudotime analysis and converting static data (alternative splicing event expression and RNA-binding protein expression) into pseudotime progression data. A hypothetical example of a gene with three splice variants is given in [22] to illustrate the alternative splicing event expression. Here, variant 1 is formed by all three exons, whereas variant 2 skips the second exon and variant 3 skips the third exon. Usually, multiple variants are expressed simultaneously at any given time. Suppose that variant 1 makes up for 60% of the overall expression of the gene, variant 2 for 30% and variant 3 for 10%. Hence, there are 3 alternative splicing events of this gene, which are recorded as A1, A2 and A3 for convenience, and the expression of A1 is the overall expression of the gene multiplied by 60%, the expression of A2 is the overall expression of the gene multiplied by 30%, and the expression of A3 is the overall expression of the gene multiplied by 10%. Meanwhile, RNA-binding protein expression refers to the gene expressions of this RNA-binding protein. Then, to determine the dynamic regulatory relationships between alternative splicing events and RNA-binding proteins, we assume that the dynamic regulatory relationships can be modeled as a dynamical system. Based on our experience, we believe that the above regulatory network is always sparsely connected. According to the prior hypothesis of sparsity, the Bayesian Lasso method is used to estimate the parameters in the dynamical system. In particular, to select EM transition-associated alternative splicing events and RNA-binding proteins, a trend analysis method is applied. Specifically, based on the obtained pseudotime progression data, for each alternative splicing event (RNA-binding protein), the linear trend divided by the detrended standard deviation is calculated, and then the absolute value of this value is taken. This absolute value is defined as the score for each alternative splicing event (RNA-binding protein). The top 50 alternative splicing events with the highest scores are considered as EM transition-associated alternative splicing events, and the top 10 RNA-binding proteins with the highest scores are considered as EM transition-associated RNA-binding proteins.

### Data preprocessing

The raw data adopted in this paper is the Processed TCGA BRCA Level 3 RNA-SeqV2 gene expression data were downloaded from the Genomic Data Commons (GDC) Legacy Archive (https://portal.gdc.cancer.gov/legacy-archive). The data and code for PCB are available at: http://hkumath.hku.hk/~wkc/PCB(data+code).zip.

Epithelial and mesenchymal tumors were then expected to be distinguished from the original TCGA-BRCA samples. The EM transition score was calculated as the gene expression difference between E-cadherin (CDH1) and Vimentin (VIM), where CDH1 and VIM are correspondingly highly expressed in epithelial and mesenchymal cell states. Based on EM transition scores, samples with EM transition scores one standard deviation above the mean were classified as mesenchymal, and samples with EM transition scores one standard deviation below the mean were classified as epithelial. This classification identified 143 epithelial samples and 157 mesenchymal samples out of a total of 1215 TCGA-BRCA samples.

Known alternative splicing events could be classified using an annotated set of splicing events provided by the splicing analysis tool mixture-of-isoforms (MISO) model downloaded from (http://hollywood.mit.edu.eproxy.lib.hku.hk/burgelab/miso/) [23]. Based on the above analysis, a comprehensive list of 15753 annotated cassette exons events was created for the 300 samples (143 epithelial samples and 157 mesenchymal samples). Meanwhile, we also collected the gene expressions of 1532 RNA-binding proteins in a recent census of human RNA-binding proteins for all breast cancer (BRCA)-associated samples [24, 25].

Now, 143 epithelial specimens and 157 mesenchymal specimens were extracted from the original large dataset, and each sample contained the expressions of 15753 alternative splicing events and the gene expressions of 1532 RNA-binding proteins (15753 × 300 data matrix and 1532 × 300 data matrix). For more details, see for instance, [17, 21].

For 15753 × 300 data matrix, there are many missing values. In order to fill in the missing data more accurately, we first deleted the row where the number of missing data is greater than or equal to 100. Then knnimptute was used to impute the missing values. For the filled data, we calculated the row variance and deleted the row whose row variance is 0. Finally, for alternative splicing events, 10049 × 300 data matrix was obtained, see S1 Table for details. Similarly, for RNA-binding proteins, 1525 × 300 data matrix was obtained, see S2 Table for details. Moreover, the pathological grading (stage I-IV) and TNM staging of the 300 specimens from TCGA were given in S3 Table.

### Pseudotime analysis

We applied the cross-sectional dataset of breast cancer patients in S2 Table, and used the pseudotime analysis to transform the static dataset into a time-series dataset.

Specifically, we performed the pseudotime progression inference to order 300 specimens based on the whole RNA-binding protein expression profile in S2 Table. Here, the epithelial and mesenchymal specimens were labeled 1 and 2, respectively. The algorithm for pseudotime analysis is described in S1 Text. Using this algorithm, for each specimen we calculated its pseudotime score. According to the pseudotime score, the corresponding specimens were arranged in ascending order, and the sorted samples were mapped to a smoothed temporal trajectory. Based on the sorted TCGA barcode, we could directly sort the alternative splicing event expression profile in S1 Table, and similarly mapped the sorted samples to a smoothed temporal trajectory.

### EM transition-associated alternative splicing event and RNA-binding protein selection

To obtain alternative splicing events and RNA-binding proteins associated with the EM transition process in breast cancer, we used a trend analysis technique. Based on the above pseudotime analysis, we define *X*_*i*_(*s*) as the smoothed expression of the alternative splicing event (or RNA-binding protein) index *i* at the inferred pseudotime progression status *s*. Each index *i* corresponds to an alternative splicing event (RNA-binding protein). For example, *i* = 1 of RNA-binding protein represents the RNA-binding protein A1CF. Since there are 10049 alternative splicing events and 1525 RNA-binding proteins, the index 1 ≤ *i* ≤ 10049 (1 ≤ *i* ≤ 1525) of alternative splicing event (RNA-binding protein). The trend analysis method is as follows:

**Algorithm 1** The trend analysis method

1: Input: smoothed expression data *X*_*i*_.

2: Fit *X*_*i*_ to a linear function *L*_*i*_ × *s* + *C*_*i*_. Here, the estimated coefficient is denoted as *L*_*i*_ and the constant is denoted as *C*_*i*_.

3: Calculate the standard deviation of the detrended expression data, and denote as *V*_*i*_. The detrended expression data *H*_*i*_(*s*) is obtained as *H*_*i*_(*s*) = *X*_*i*_(*s*) − (*L*_*i*_ × *s* + *C*_*i*_), which means that removes the best straight-line fit linear trend from *X*_*i*_. Therefore, *V*_*i*_ = *std*(*H*_*i*_(*s*)).

4: A score is defined, *R*_*i*_ = |*L*_*i*_/*V*_*i*_|.

5: Output: score *R*_*i*_.

Linear trend represents a systematic increase or decrease in data over time. By removing the linear trend from the data, the fluctuation of the detrended data was considered, and such fluctuation is not caused by time change. Therefore, we believe that the higher the score *R*_*i*_, the more likely the expression of the corresponding alternative splicing event *i* (RNA-binding protein *i*) changes significantly in the EM transition process, in other words, this alternative splicing event *i* (RNA-binding protein *i*) is more likely to be EM transition-related. An example is given in S2 Text to clarify this method.

For each alternative splicing event 1 ≤ *i* ≤ 10049 (RNA-binding protein 1 ≤ *i* ≤ 1525), we can get a score according to the above algorithm. We selected alternative splicing events and RNA-binding proteins with high scores.

### Identification of the regulatory relationships between alternative splicing events and RNA-binding proteins

As described in [13], sequence-specific RNA-binding proteins bind to pre-mRNA to control alternative splicing. And each alternative splicing event is controlled by multiple RNA-binding proteins. Based on the mass action kinetics [20, 26, 27] and the above priori knowledge, the regulatory relationships between alternative splicing events and RNA-binding proteins can be described by means of the following dynamical system,

$$\begin{array}{c} \frac{dX_{i}\left(s\right)}{ds}=\sum_{j\neq i}a_{ij}X_{i}\left(s\right)\cdot X_{j}\left(s\right)+\sum_{l=1}^{M}b_{il}X_{i}\left(s\right)\cdot U_{l}\left(s\right)-d_{i}X_{i}\left(s\right), \end{array}$$ (1)

$$\begin{array}{c} \frac{dU_{l}\left(s\right)}{ds}=\sum_{k\neq l}c_{lk}U_{l}\left(s\right)\cdot U_{k}\left(s\right)-d_{l}^{\prime }U_{l}\left(s\right). \end{array}$$ (2)

Here *X*_*i*_(*s*) and *U*_*l*_(*s*) represent the expression level of alternative splicing event *i*, *i* = 1, 2, …, *N* and RNA-binding protein *l*, *l* = 1, 2, …, *M* in breast cancer with pseudotime progression status *s*, respectively. Moreover, *a*_*ij*_ is the dynamic regulatory coefficient from alternative splicing event *j* to alternative splicing event *i*, where *i* ≠ *j* and *i*, *j* = 1, 2, …, *N*; *b*_*il*_ is the dynamic regulatory coefficient from RNA-binding protein *l* to alternative splicing event *i*, where *i* = 1, 2, …, *N* and *l* = 1, 2, …, *M*; *c*_*lk*_ is the dynamic regulatory coefficient from RNA-binding protein *k* to RNA-binding protein *l*, where *l* ≠ *k* and *l*, *k* = 1, 2, …, *M*, and *d*_*i*_ is the self-degradation rate of alternative splicing event *i*; $d_{l}^{\prime }$ is the self-degradation rate of RNA-binding protein *l*. Since alternative splicing events rely on RNA-binding proteins, but not the other way around, Eq (1) has a middle term and Eq (2) has no middle term. For more details on this dynamical system, see S3 Text. Notably, the dynamical system can be reasonably assumed to be sparsely connected.

Then, the Bayesian Lasso method is applied to estimate parameters of the above model. Although the Bayesian method is typically employed with a Gaussian prior, which allows for simple analysis and processing due to conjugation, the sparsity cannot be guaranteed using Gaussian prior. As mentioned in [28], when sparsity is taken as a key prior assumption, the Laplace distribution prior is often considered, and because of its log convexity, it also has a great computational advantage. In addition, in the next section, we also compare the Bayesian lasso method with the regular Bayesian method to further illustrate that the Bayesian Lasso method is more applicable here. The Markov chain Monte Carlo (MCMC) algorithm is applied to sample from the posterior. In this study, if the 95% credible interval (CI) of the parameter estimated by *a*_*ij*_ (*b*_*il*_) does not contain zero, it is considered that there is a directed edge from alternative splicing event *j* (RNA-binding protein *l*) to alternative splicing event *i*; otherwise, the edge is regarded as non-existent. Similarly, we can determine the directed edge from RNA-binding protein *k* to RNA-binding protein *l*. See S3 Text for details.

## Results and discussions

### Testing PCB with a synthetic dataset

**Fig 2:**
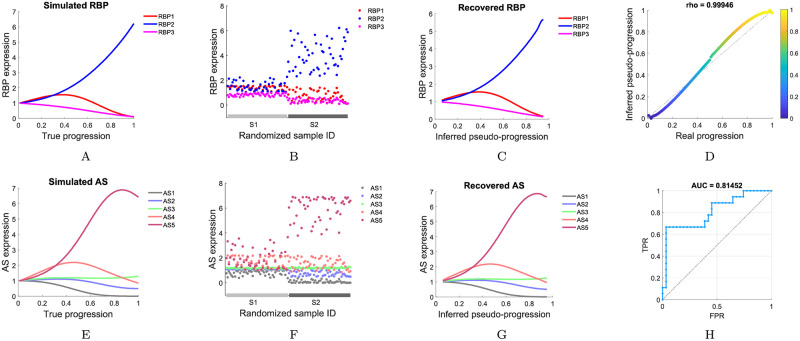
Illustrating the function of our proposed model using a synthetic dataset. A set of expression data for 3 RNA-binding proteins (RBPs) and 5 alternative splicing (AS) events in 100 cancer specimens was simulated. (A) and (E) Original RNA-binding protein (alternative splicing event) expression data. (B) and (F) Simulated cross-sectional RNA-binding protein (alternative splicing event) expression data. The sample IDs of the synthetic data were randomized and the EM transition process information was retained. (C) and (G) Recovered RNA-binding protein (alternative splicing event) expression dynamics according to inferred pseudotime progression trajectory. (D) Comparison of the inferred pseudotime progression with the true progression in the synthetic dataset, evaluated using Spearman’s rank correlation coefficient (rho). (H) Accuracy of the dynamical systems inference evaluated using the AUC of ROC.

To evaluate the effectiveness and accuracy of our proposed model PCB, a set of synthetic cross-sectional expression data was generated (S4 Text). In order to visualize the result, 3 RNA-binding proteins and 5 alternative splicing events in 100 cancer specimens were considered (Fig 2A and 2E). Based on the sample-randomized RNA-binding protein expression profile (Fig 2B), we evaluated whether the proposed method could accurately order the samples. Based on the sorted sample data, we further evaluated whether the proposed method could effectively reconstruct the regulatory relationships between alternative splicing events and RNA-binding proteins.

First, we calculated the pseudotime score of each specimen and used this score to sort the samples. Comparing the inferred pseudotime score of each specimen with the true progression (Fig 2D), the proposed model correctly recovered the real order of samples (Spearman’s rho = 0.99946). Moreover, along with the pseudotime progression, both RNA-binding protein expression dynamics (Fig 2C) and alternative splicing event expression dynamics (Fig 2G) showed contours that are very similar to the initial data. Then, by the Bayesian Lasso method, we reconstructed the regulatory relationships between alternative splicing events and RNA-binding proteins. Compared with the real dynamical system, the area under the curve (AUC) of receiver operating characteristic (ROC) with/without interactions was used to assess the accuracy of the dynamical system inference. Here, the AUC value reached 81.452% (Fig 2H).

**Fig 3:**
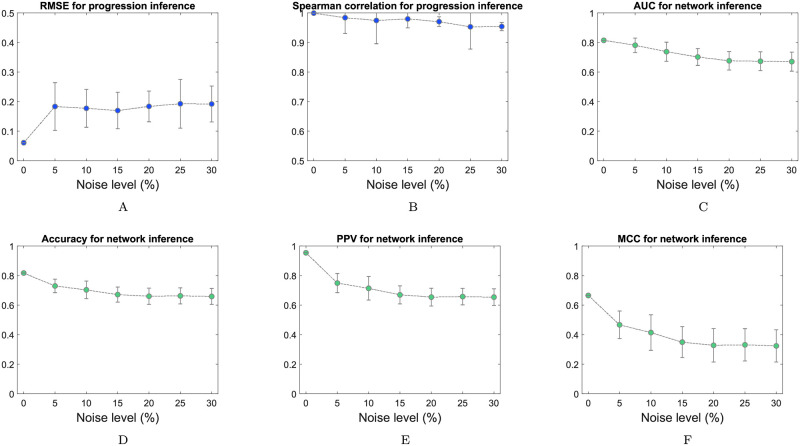
Evaluation metrics were used to verify the robustness of PCB against a series of variations in the data using multiplicative exponential noise (with CVs ranging from 0% to 30%). (A) and (B) The root mean square error (RMSE) and Spearman correlation coefficients were employed to evaluate the accuracy of the temporal progression inference. (C),(D),(E) and (F) The AUC, accuracy, positive predictive value (PPV) and Matthews correlation coefficient (MCC) were employed to evaluate the robustness of the reconstructed regulatory relationships between alternative splicing events and RNA-binding proteins.

The robustness of the model to the measured variables in the data was illustrated by numerical verification and mathematical proof. First, multiplicative exponential noise was used to randomly perturb both alternative splicing event and RNA-binding protein expressions to simulate diverse levels of measurement variabilities in the data, which resulted in a series of coefficients of variation (CVs). Here, we assumed that noise is generated from an exponential distribution with a mean *μ*, where 0% ≤ *μ* ≤ 10%. Sample IDs were randomly assigned to simulate sample-based snapshots of alternative splicing event expression data (RNA-binding protein expression data), but still retained each specimen’s EM transition process information. PCB was applied to infer the regulatory relationships of each dataset. Evaluation metrics were used to verify the robustness of PCB against a series of changes in the data (with CVs ranging from 0% to 30%) (Fig 3). Moreover, the robustness of the model to the measured variables in the data was illustrated as follows.

**Theorem 1** *Suppose there are two trajectories of pseudotime progression* *s*(*r*) and $\tilde{s}\left(r\right)$ *with the same root* *r* ∈ *I* = [0, 1]. *Define* $\|\tilde{s}{-s\|}_{L^{2}}=(\int_{I}|\tilde{s}-s{{|}^{2}dr)}^{1/2}$. *If* (*X*_*i*_(*s*), *U*_*l*_(*s*), *a*_*ij*_, *b*_*il*_, *c*_*lk*_) *and* $\left(X_{i}\left(\tilde{s}\right),U_{l}\left(\tilde{s}\right),{\tilde{a}}_{ij},{\tilde{b}}_{il},{\tilde{c}}_{lk}\right)$ *both satisfy the equations of the pseudotime progression-dependent dynamical system, i.e.*,

$$\begin{array}{c} \frac{dX_{i}\left(s\right)}{ds}=\sum_{j\neq i}a_{ij}X_{i}\left(s\right)\cdot X_{j}\left(s\right)+\sum_{l=1}^{M}b_{il}X_{i}\left(s\right)\cdot U_{l}\left(s\right)-d_{i}X_{i}\left(s\right), \end{array}$$ (3)

$$\begin{array}{c} \frac{dU_{l}\left(s\right)}{ds}=\sum_{k\neq l}c_{lk}U_{l}\left(s\right)\cdot U_{k}\left(s\right)-d_{l}^{\prime }U_{l}\left(s\right), \end{array}$$ (4)

$$\begin{array}{c} \frac{dX_{i}\left(\tilde{s}\right)}{d\tilde{s}}=\sum_{j\neq i}{\tilde{a}}_{ij}X_{i}\left(\tilde{s}\right)\cdot X_{j}\left(\tilde{s}\right)+\sum_{l=1}^{M}{\tilde{b}}_{il}X_{i}\left(\tilde{s}\right)\cdot U_{l}\left(\tilde{s}\right)-{\tilde{d}}_{i}X_{i}\left(\tilde{s}\right), \end{array}$$ (5)

$$\begin{array}{c} \frac{dU_{l}\left(\tilde{s}\right)}{d\tilde{s}}=\sum_{k\neq l}{\tilde{c}}_{lk}U_{l}\left(\tilde{s}\right)\cdot U_{k}\left(\tilde{s}\right)-{\tilde{d}}_{l}^{\prime }U_{l}\left(\tilde{s}\right), \end{array}$$ (6)

*where* *i* = 1, 2, …, *N* *and* *l* = 1, 2, …, *M*. *Then we have*

$$\begin{array}{c} \underset{\|\tilde{s}{-s\|}_{L^{2}}\to 0}{\text{lim}}(\sum_{j=1}^{N}{\left({\tilde{a}}_{ij}-a_{ij}\right)}^{2}+\sum_{l=1}^{M}{\left({\tilde{b}}_{il}-b_{il}\right)}^{2})=0, \end{array}$$ (7)

*and*

$$\begin{array}{c} \underset{\|\tilde{s}{-s\|}_{L^{2}}\to 0}{\text{lim}}(\sum_{k=1}^{M}{\left({\tilde{c}}_{lk}-c_{lk}\right)}^{2})=0. \end{array}$$ (8)

The details of mathematical proof can be found in S5 Text.

By testing PCB with a synthetic dataset, we found that both the pseudotime progression trajectory and network structure of the dynamic regulatory relationship model inferred by PCB were quite robust to the variation in the data (Figs 2 and 3).

### Identifying EM transition-associated alternative splicing events and RNA-binding proteins during breast cancer progression

The model was applied to a dataset of breast cancer from [21]. To find alternative splicing events and RNA-binding proteins that were strongly associated with the EM transition process, we first used pseudotime analysis to obtain the ordered RNA-binding protein (alternative splicing event) expression data. The sorted specimens are shown in S4 Table. Here we find that after the pseudotime analysis, all samples of epithelial state are at the beginning of the trajectory, and all samples of the mesenchymal state are at the tail of the trajectory, and most of the samples at the beginning of the trajectory have no distal metastasis (M0). However, this order seems not to be related to the pathological stage. Then using Algorithm 1, we calculated the score of each RNA-binding protein (alternative splicing event). Finally, according to the scores obtained, we selected the top 50 alternative splicing events and the top 10 RNA-binding proteins (see for more details in S5 Table).

We found that most of these 10 RNA-binding proteins increased during the EM transition, and only LARP4 decreased (see S1 Fig for details). To confirm that the RNA-binding proteins we selected were indeed related to EM transition in breast cancer, we consulted relevant literature. Among the 10 RNA-binding proteins we identified, SMAD6 and PIWIL4 have been confirmed in the literature that they were related to EM transition in breast cancer (see details in Table 1), and the roles of these two RNA-binding proteins in the EM transition process (activation or inhibition) were consistent with our reconstructed expression dynamics (S1 Fig). Additionally, ZFP36, LARP4 and ZCCHC24 have been confirmed to be associated with breast cancer metastasis and invasion (see details in Table 1). Moreover, in [29–34], it was also mentioned that the seven RNA-binding proteins (ZFP36, ZCCHC24, ZFP36L2, TDRD10, PTRF, CSDA and DZIP1) were related to the EM transition process, although these studies did not evaluate the EM transition process in breast cancer. Therefore, eight of the RNA-binding proteins we identified (ZFP36, LARP4, ZCCHC24, ZFP36L2, TDRD10, PTRF, CSDA, and DZIP1) could provide biologists with a new set of RNA-binding proteins to test the regulatory role of the EM transition process in breast cancer.

**Table 1:** RNA-binding proteins (RBPs) reported in previously published literature.

RBP	EM transition regulation	Description of RBPs role in breast cancer and relevant references
ZFP36	Activator	In vivo and in vitro, NNT-AS1 knockout can inhibit the progression and metastasis of breast cancer by interacting with ZFP36 [36].
LARP4	Inhibitor	LARP4 the inhibits migration and invasion of cancer cells [37].
SMAD6	Activator	[38] confirmed the importance of BMP signaling in the EM transition process and in vivo invasion. Overexpression of SMAD6 in the estrogen receptor breast cancer cell lines can effectively block BMP signalling.
ZCCHC24	Activator	ZCCHC24 seems to change the structure of breast cancer cells, increasing the invasiveness of the fatal disease [39].
PIWIL4	Activator	PIWIL4 is necessary for the EM transition process [40]. PIWIL4 is a novel estrogen receptor signaling regulator that can be targeted to inhibit the growth and migration of breast cancer [41].

**Fig 4:**
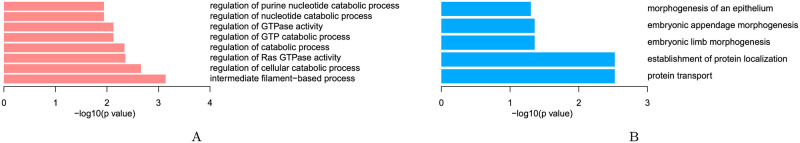
Network ontology of corresponding genes for 50 alternative splicing events. (A) Corresponding ascending genes. (B) The corresponding descending genes.

Then, for the top 50 alternative splicing events, a heatmap with hierarchical clustering was shown in S2 Fig. Moreover, for these two groups of alternative splicing events, we analyzed the network ontology of their corresponding genes using NOA software [35] (see details in Fig 4), which showed that the alternative splicing events we found were related to EM transition [42–44]. For example, [43] mentioned that intermediate filaments plays a vital role in EM transition. Ras GTPase-activating protein SH3 domain binding protein 1 (G3BP1) is an important Ras mediator and [44] indicated that upregulation of G3BP1 promotes the EM transition process in breast cancer cells.

Moreover, we used dbEMT [45] (a literature based genetic resource for exploring EM transition related human genes) to search the corresponding genes for 50 alternative splicing events, and found that seven of them (MAP4K4, NUMB, CTNND1, NF1, FGFR1, MAP3K7, EXOC7) were clearly identified as EM transition-related genes. In addition, we found that about 23 corresponding genes were mentioned to be related to the EM transition process in the literature (See Table 2 for details).

**Table 2:** Genes reported in previously published literature.

Gene	EM transition regulation	Description of gene role in the EM transition (EMT) process and relevant references
MAP4K4	Activator	MAP4K4 primarily promotes the EMT process and invasiveness of hepatocellular carcinoma cells by activating JNK and NF-kappaB signaling [50].
ERP29	Inhibitor	ERP29 negatively regulates the EMT process in breast cancer cells [51].
NUMB	Inhibitor	By activating the Notch signaling, the deletion of NUMB promotes the EMT program in triple-negative breast cancer cells [52].
ARNT	Inhibitor	ARNT, as a inhibitor, prevents the progression of EMT by reducing the expression of metastasis related gene [53].
CTNND1	Inhibitor	Through E-cadherin, CTNND1 may regulate EMT in oral squamous cell carcinoma [54].
TCF12	Activator	TCF12 induces the EMT process in hepatocellular carcinoma cells [55].
KIAA1217	Activator	KIAA1217 significantly promotes cell migration and invasion by inducing the EMT process in vitro [56].
EPB41	Inhibitor	Knockout of EPB41 with shRNAs can induce morphological changes associated with the EMT process in non-small-cell lung cancer PC9 or A549 cells [57].
CLSTN1	Inhibitor	A CLSTN1 short isoform can inhibit EMT in breast cancer cells [58].
NF1	Inhibitor	Neurofibromin, encoded by NF1 gene, may inhibit the EMT process [59].
FGFR1	Activator	Activation of FGFR1 can promote the EMT process in breast cancers [60].
LRRFIP2	Activator	The WNT pathway is another signaling pathway that crosstalks the TGF-*β* pathway and promotes EMT, and LRRFIP2 is an activator of canonical WNT signaling [61, 62].
SPTAN1	Activator&Inhibitor	SPTAN1 interacts with ankyrin, E-cadherin, and Na/K-ATPase. Therefore, it may affect the EMT process and metastasis [63].
PLOD2	Activator	PLOD2 promotes the EMT process, which is an upstream factor regulating the PI3K/Akt signaling pathways in glioma cells [64].
MAP3K7	Activator	Inhibition of MAP3K7 can block the EMT process in vitro [65].
MBNL1	Activator&Inhibitor	Knockout of MBNL1 induces EMT-like morphological changes in the HCT-116 cells while enhancing cell motility, upregulating Snail expression, and downregulating E-cadherin. In contrast, the ectopic overexpression of MBNL1 inhibits EMT, which is characterized by E-cadherin upregulation and Snail expression downregulation [66].
SPAG9	Activator	SPAG9 regulates the EMT process in bladder transitional carcinoma [67].
MACF1	Activator	MACF1 knockout induces the conversion of mesenchymal and epithelial biomarkers expression. Expression levels N-cadherin and TGF*β* are significantly reduced, while expression levels of E-cadherin and SMAD-7 are elevated, suggesting that MACF1 has influence on the EMT process in this particular case [68].
EXOC7	Inhibitor	The expression of EXOC7 epithelial isoform can affect the levels of key EMT transcriptional regulators and is sufficient to drive the transition to epithelial phenotypes [69].
PBX1	Activator	PBX1 promotes EMT induction in lung cancer [70].
MYOF	Activator	The loss of MYOF shifts the movement of breast cancer cells towards collective migration and promotes changes in the shape of the mesenchymal-to-epithelial [71, 72].
GIT2	Inhibitor	Loss of GIT2 induces the EMT process by promoting the expression of Zeb1 [73].
NCOR2	Inhibitor	Reduce the activation of Notch signaling pathway by upregulating NCOR2 and Notch has important function on promoting the development of EMT and tumor progression [74, 75].

**Fig 5:**
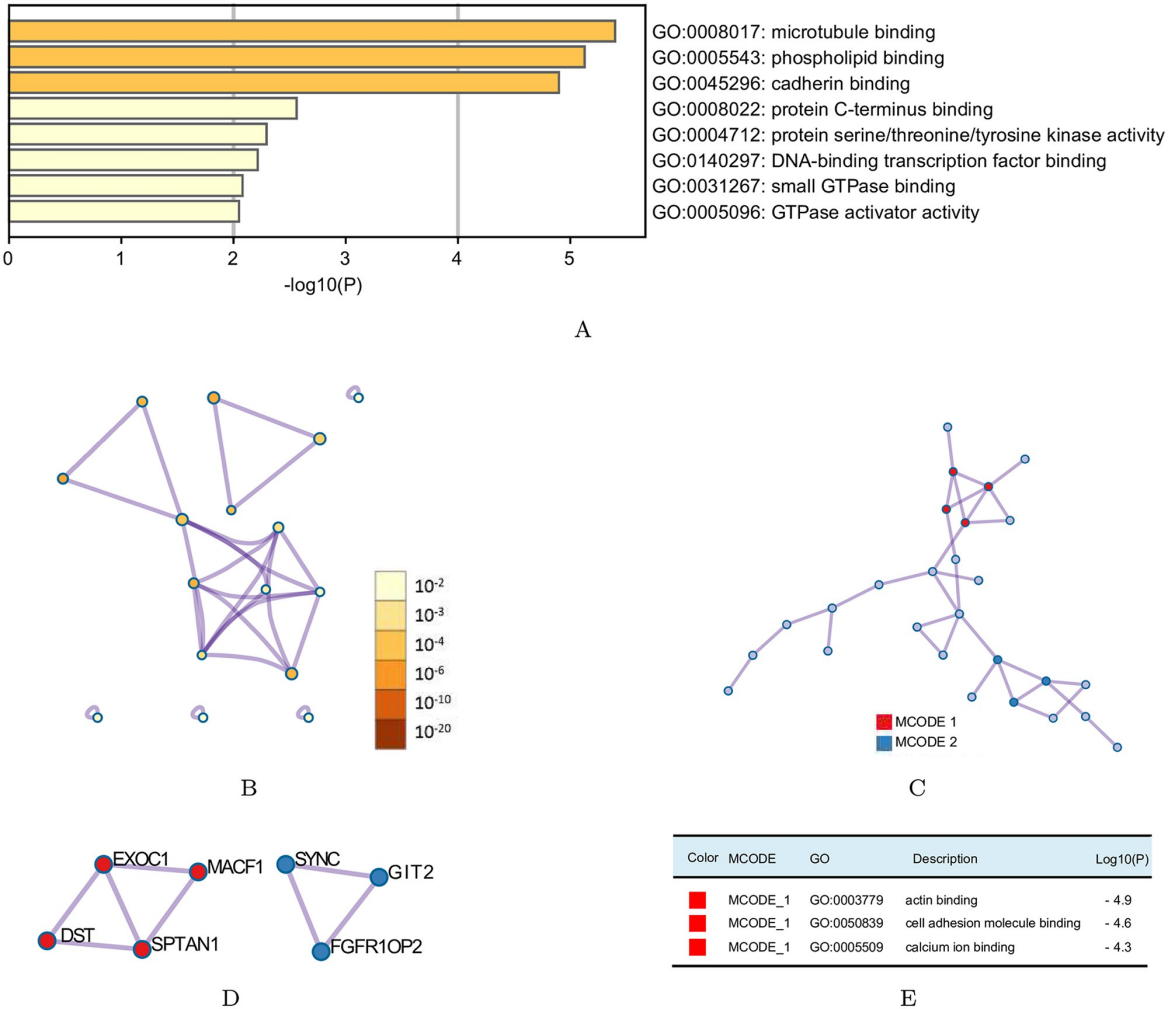
Enrichment analysis of the marker genes identified by PCB (Metascape). (A) Pathway and process enrichment analysis has been carried out with Gene Ontology (GO) Molecular Functions. Bar graph of enriched terms across the marker genes, colored by *p*-values. (B) Network of enriched terms across the marker genes: colored by *p*-value, where terms containing more genes tend to have a more significant *p*-value. (C) Protein-protein interaction network and the MCODE component identified in the marker genes. (D) Pathway and process enrichment analysis has been applied to the MCODE component. The three best-scoring terms by *p*-value have been retained as the functional description of the MCODE components, shown in Table (E).

To understand the biological role and potential functions of the 50 alternative splicing events, functional enrichment analysis of the corresponding genes was conducted using Metascape (https://metascape.org). Pathway and process enrichment analysis has been carried out with Gene Ontology (GO) Molecular Functions. All genes in the genome have been used as the enrichment background. Terms with a *p*-value <0.01, a minimum count of 3, and an enrichment factor >1.5 were collected and grouped into clusters based on their membership similarities. The results of GO analysis were displayed in Fig 5A and 5B. And significant modules of Protein-protein interaction enrichment analysis (PPI) were identified in Fig 5. Furthermore, the molecular complex detection (MCODE) algorithm [46] was applied to analyze clusters of the PPI networks (Fig 5D and 5E). We could see from Fig 5 that the main biological functions of the 50 alternative splicing candidate genes focused on binding with microtubule, phospholipid, cadherin, protein C-terminus, small GTPase and DNA-binding transcription factor, protein serine/threonine/tyrosine kinase activity and GTPase activator activity. PPI showed that the interaction between the 27 alternative splicing events among the 50 alternative splicing events were recruited on cell adhesion molecule binding, cadherin binding and structural molecule activity. Two molecular complexes were detected by the MCODE algorithm. Complex 1, including SPTAN1, EXOC1, MACF1 and DST, was involved in actin binding, cell adhesion molecule binding and calcium ion binding. Therefore, the above analysis revealed that the top 50 alternative splicing events mainly regulated the EM transition process by affecting cytoskeletal rearrangement and cell adhesion. Since, the changes of cytoskeletal and cell adhesion are the key characteristics in the EM transition process [47–49], suggesting that the 50 alternative splicing events we got is crucial for the regulation of the EM transition process, and thus also proves the reliability of our calculating method.

The above evidence shows that our method effectively selected alternative splicing events and RNA-binding proteins related to EM transition.

**Fig 6:**
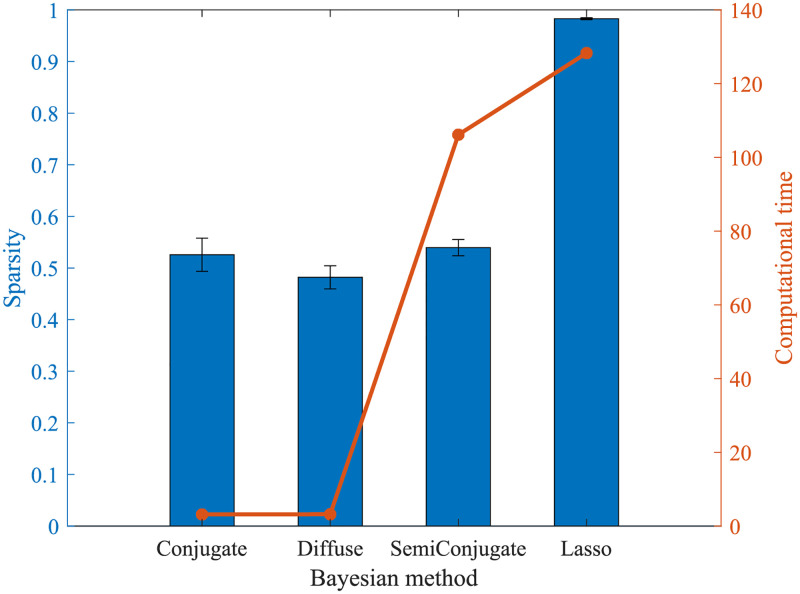
Computational time and sparsity of Bayesian Lasso method and other Bayesian methods.

We then inferred the dynamic regulatory relationships of the above 50 alternative splicing events and 10 RNA-binding proteins. Here, we first compare the Bayesian Lasso method with other Bayesian methods (Bayesian Conjugate, Bayesian SemiConjugate, and Bayesian Diffuse) in estimating the parameters of the dynamical system (Eqs (1) and (2)), mainly considering computational time and sparsity. We obtained the computational time of parameter estimates for two dynamical systems (epithelial state and mesenchymal state) and found that the computational time was not long. Specifically, Bayesian Conjugate method and Bayesian Diffuse method are about 3 seconds, Bayesian SemiConjugate is about 106 seconds, and Bayesian Lasso method is about 128 seconds. The sparsity = 1 − |interactions|/(*N*^2^ + *NM* + *M*^2^), where *N* is the number of alternative splicing events and *M* is the number of RNA-binding proteins, which refers to the degree to which parameters contain zero values (no interaction). For each Bayesian method, we still considered the 95% CI of the parameter estimated and we calculated the sparsity of the parameters estimated by the dynamical system in the epithelial state and the sparsity of the parameters estimated by the dynamical system in the mesenchymal state. According to Fig 6, using other Bayesian methods (Bayesian Conjugate, Bayesian SemiConjugate, and Bayesian Diffuse) cannot guarantee sparsity, but the dynamical system is sparsely connected according to prior knowledge.

Therefore, despite the computational time is longer, we still used Bayesian Lasso method to estimate parameters of the dynamical system (Eqs (1) and (2)).

Based on (2), we first analyzed the mutual regulatory relationships between 10 RNA-binding proteins and obtained two RNA-binding protein networks (S3 Fig). We found that the dynamic regulatory relationships between these 10 RNA-binding proteins were completely different in the epithelial state and the mesenchymal state (see S6 Table for details). Subsequently, we used the Maximal Clique Centrality (MCC) method proposed by [76] to rank the nodes in the two RNA-binding protein networks, respectively. Based on the MCC method, ZCCHC24 was considered the most influential RNA-binding protein in the RNA-binding protein network during the epithelial state (see S7 Table for details) and PIWIL4 was considered as the most influential RNA-binding protein in the RNA-binding protein network during the mesenchymal state (see S7 Table for details). Notably, [40] confirmed that MDA-MB-231 cells lacking PIWIL4 abandoned the mesenchymal cell fate and regained the key characteristics of epithelial cell fate. Moreover, PIWIL4 is highly expressed in the cytoplasm of MDA-MB-231 cells derived from breast cancer. PIWIL4 is necessary for the transformation of epithelial cells into mesenchymal cells and the migration ability of MDA-MB-231 cells.

**Fig 7:**
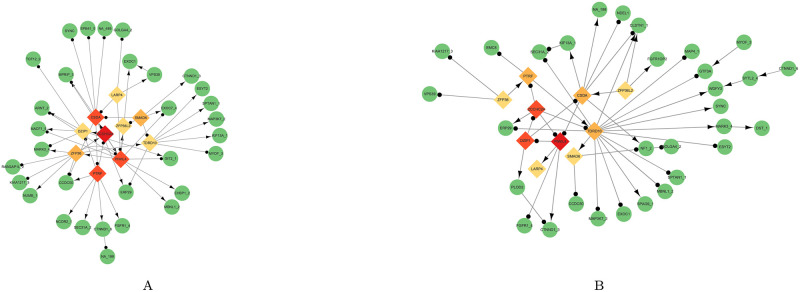
Dynamic regulatory relationship between 50 alternative splicing events and 10 RNA-binding proteins. (A) Dynamic regulatory relationships between 50 alternative splicing events and 10 RNA-binding proteins in the epithelial state. ZCCHC24 was identified as the most influential RNA-binding protein. (B) Dynamic regulatory relationships between 50 alternative splicing events and 10 RNA-binding proteins in the mesenchymal state. PIWIL4 was identified as the most influential RNA-binding protein.

Based on (1), we analyzed the mutual regulatory relationships between 50 alternative splicing events and 10 RNA-binding proteins, and we obtained two alternative splicing regulatory networks, respectively. We found that an alternative splicing event could be regulated by multiple RNA-binding proteins, and when multiple RNA-binding proteins affect an alternative splicing event, these RNA-binding proteins play an antagonistic or coordinating role. For example, in the epithelial state, three RNA-binding proteins (CSDA, ZFP36 and PTRF) jointly regulate the alternative splicing event CCDC50, in which CSDA and ZFP36 inhibit the expression of CCDC50, wherea PTRF promotes the expression of CCDC50. However, one RNA-binding protein can also affect multiple alternative splicing events. For example, in the mesenchymal state, the RNA-binding protein TDRD10 is associated with multiple alternative splicing events. Finally, we obtained Fig 7 by integrating RNA-binding protein network information with alternative splicing regulatory network information. We found that PIWIL4 and TDRD10 have the largest out-degree values in the epithelial and mesenchymal states, respectively.

Therefore, the above analysis revealed that ZCCHC24, PIWIL4, and TDRD10 may be key RNA-binding proteins in EM transition of breast cancer. In particular, [40, 41] confirmed that PIWIL4 was related to EM transition in breast cancer.

### Regulation of alternative splicing events of the CD44 gene in breast cancer

The CD44 pre-mRNA contains 19 exons, nine of which are alternatively spliced. Recent studies confirmed that alternative splicing events of the CD44 gene have a causal relationship to the EM transition process and breast cancer metastasis [7, 9, 77, 78]. By studying the regulation mechanism of alternative splicing events of the CD44 gene, the antagonism between hnRNPM and ESRP1 has also been determined. Numerous studies have shown that hnRNPM promotes CD44 variable exon skipping, which is conducive to forming the mesenchymal phenotype, whereas ESRP1 stimulates the CD44 variable exon inclusion body and promotes an epithelial cellular state [7, 9, 77, 79]. Furthermore, in [8], the RNA-binding proteins (ESRP1 and ESRP2) were identified as key regulators of splicing events related to the EM transition process. In this part, we analyzed alternative splicing events of the CD44 gene coregulated by hnRNPM, ESRP1 and ESRP2.

**Fig 8:**
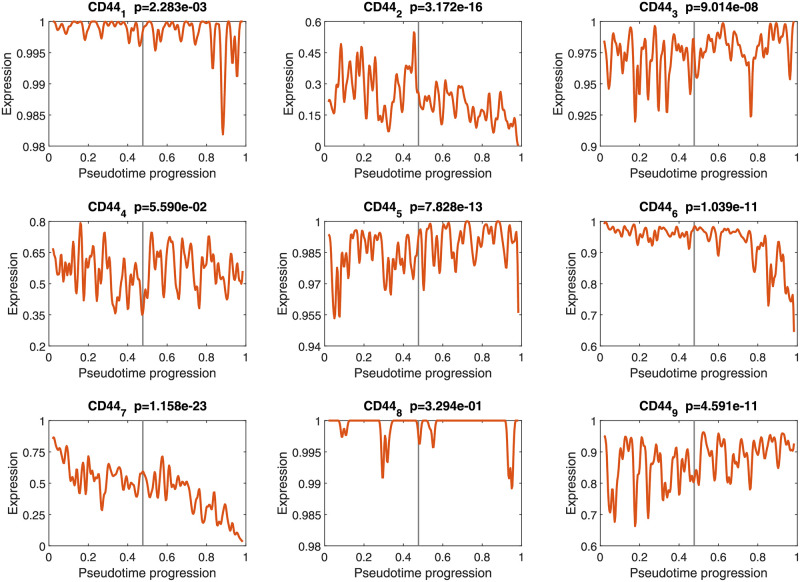
Reconstructed expression dynamics of alternative splicing events of the CD44 gene.

**Fig 9:**
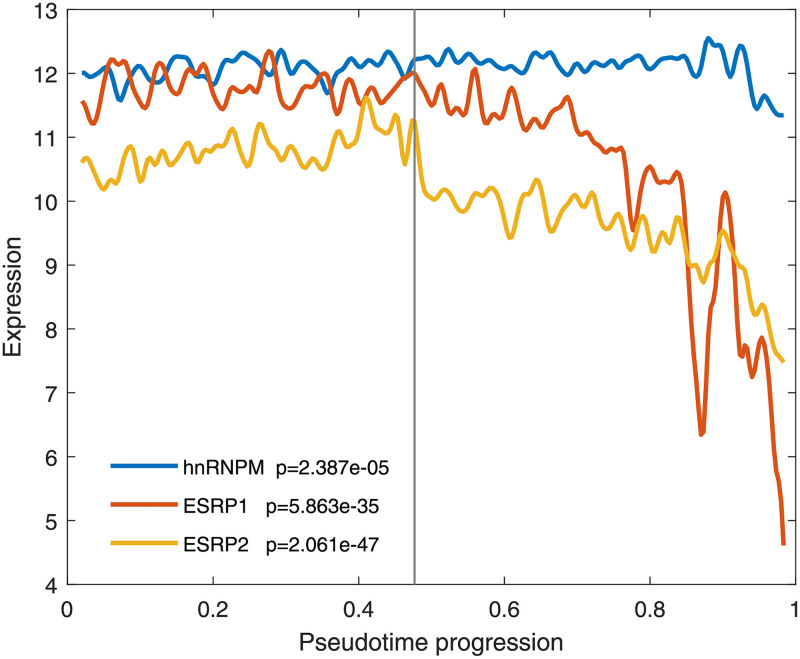
Reconstructed expression dynamics of hnRNPM, ESRP1 and ESRP2.

We still applied the model to the dataset from [21]. The pseudotime progression inference was first performed. To identify the dynamic regulatory relationships between alternative splicing events and RNA-binding proteins, we collected nine alternative splicing events of the gene CD44 and three RNA-binding proteins (hnRNPM, ESRP1 and ESRP2). We obtained the reconstructed expression dynamics of nine alternative splicing events of the CD44 gene and the reconstructed expression dynamics of hnRNPM, ESRP1 and ESRP2 (see details in Figs 8 and 9), respectively. The Wilcoxon rank-sum test (two-tailed) *p*-values were calculated to evaluate statistical significance. The *p* values <0.05 were considered significant.

Here, we found the *p*-values of CD44_4_ and CD44_8_ were greater than 0.05. Therefore, when we reconstructed the alternative splicing regulatory networks, we only considered CD44_1_, CD44_2_, CD44_3_, CD44_5_, CD44_6_, CD44_7_, and CD44_9_. In the epithelial and mesenchymal samples, we obtained the corresponding reconstructed regulatory relationships between alternative splicing events and RNA-binding proteins (all adjacency matrices are given in S8 Table).

According to Fig 9 and the reconstructed regulatory relationships between alternative splicing events and RNA-binding proteins in epithelial and mesenchymal samples, we found that hnRNPM was ubiquitously expressed during the EM transition, but played a role in the state of mesenchymal cells to regulate the alternative splicing events of the CD44 gene. This finding is consistent with the result in [77]. In addition, both ESRP1 and ESRP2 were down-regulated during the EM transition process. More specifically, the expression levels of both ESRP1 and ESRP2 were high in precancerous lesions and carcinoma in situ, but their expressions were down-regulated during invasion. This phenomenon is consistent with previous experimental results [80]. These findings indicate that down-regulation of ESRP1 and ESRP2 is closely related to the motile phenotype of cancer cells.

According to Fig 8, the expression levels of the alternative splicing events CD44_2_, CD44_6_ and CD44_7_ were down-regulated. In particular, the expression levels of CD44_6_ and CD44_7_ were significantly down-regulated during the EM transition. However, the alternative splicing events CD44_5_ and CD44_9_ were up-regulated in this process. These phenomena may be related to the transformation of CD44 expression from variant subtype (CD44v) to standard subtype (CD44s) during the EM transition process [7], which is worthy of further study.

## Conclusions

Previous studies have focused on transcriptional regulation of EM transition. However, the role of global dynamic alternative splicing regulation in EM transition remains relatively uncharacterized. It is well known that a comprehensive understanding of alternative splicing and its regulation is essential for understanding its biological impact on the disease process. In this study, we attempted to characterize the alternative splicing differences and its dynamic regulation between epithelial and mesenchymal breast tumors from TCGA. To study these regulatory relationships, a PCB model was proposed. First, the time-series data of alternative splicing expression and RNA-binding protein expression were obtained by pseudotime analysis. Then, a trend analysis technique was used to select EM transition-associated alternative splicing events and RNA-binding proteins. According to the selected alternative splicing events and RNA-binding proteins, we used a dynamical system to simulate the dynamic regulatory relationships between alternative splicing events and RNA-binding proteins, and used the Bayesian Lasso method to estimate the parameters in the dynamical system. Finally, we analyzed the dynamic regulatory relationships between alternative splicing events and RNA-binding proteins, and identified the key RNA-binding proteins related to EM transition. PCB is a novel and effective tool based on dynamical system representation of alternative splicing events and RNA-binding proteins interactions during cancer progression. PCB can be used to generate experimentally testable hypotheses and to identify network-based alternative splicing biomarkers for predicting cancer prognosis and treatment response. Besides cross-sectional bulk transcriptomic data, our method could also be used to time-course scRNA-seq data. Clinical transcriptomic data of cancer patients provide an alternative way to infer alternative splicing regulation networks underlying cancer progression. Our study also sheds light on facilitating the regulatory network-based approach to identify target alternative splicing events or key RNA-binding proteins for the diagnosis or treatment of cancers.

## Supporting information

S1 Fig Reconstructed expression dynamics of 10 selected RNA-binding proteins. Most of these 10 RNA-binding proteins increased during the EM transition, and only LARP4 decreased.
(PDF)

S2 Fig A heatmap with hierarchical clustering of 50 alternative splicing events. 50 alternative splicing events were clearly clustered into two groups: a descending group and an ascending group.
(PDF)

S3 Fig The mutual regulatory relationships between 10 RNA-binding proteins. The MCC value was calculated for each RNA-binding protein. The larger the MCC value, the darker the color in the figure. (A) RNA-binding protein network in the epithelial state. Here, ZCCHC24 was identified as the most influential RNA-binding protein. (B) RNA-binding protein network in the mesenchymal state. Here, PIWIL4 was identified as the most influential RNA-binding protein.
(PDF)

S1 Table 10049 × 300 data matrix, 10049 annotated alternative splicing events for the 300 samples (143 epithelial samples and 157 mesenchymal samples). (XLSX)

S2 Table 1525 × 300 data matrix, 1525 RNA-binding proteins for the 300 samples (143 epithelial samples and 157 mesenchymal samples). (XLSX)

S3 Table 300 specimens’ pathological classification (Stage I-IV) and TNM stage. (XLSX)

S4 Table The ordered specimens. (XLSX)

S5 Table The 50 alternative splicing events and 10 RNA-binding proteins selected in this study. (XLSX)

S6 Table The dynamic regulatory relationships between the 10 RNA-binding proteins in the epithelial state and the mesenchymal state, respectively. (XLSX)

S7 Table The MCC of the 10 RNA-binding proteins in the epithelial state and the mesenchymal state, respectively. ZCCHC24 was identified as the most influential RNA-binding protein in the RNA-binding protein network during the epithelial state and PIWIL4 was identified as the most influential RNA-binding protein in the RNA-binding protein network during the mesenchymal state.
(XLSX)

S8 Table The reconstructed regulatory relationships between alternative splicing events of the CD44 gene and RNA-binding proteins (hnRNPM, ESRP1 and ESRP2). (XLSX)

S1 Text The algorithm for pseudotime analysis. (PDF)

S2 Text An example for trend analysis. (PDF)

S3 Text The model of the regulatory relationships between alternative splicing events and RNA-binding proteins. (PDF)

S4 Text Testing the model with a synthetic dataset. (PDF)

S5 Text Mathematical proof about the robustness of model to the measured variables. (PDF)
