# Supplementary figures and images for: PCB: A pseudotemporal causality-based Bayesian approach to identify EMT-associated regulatory relationships of AS events and RBPs during breast cancer progression
Source: PLoS Comput Biol. 2023 Mar 17;19(3):e1010939. doi: 10.1371/journal.pcbi.1010939 (PMC10057809; doi:10.1371/journal.pcbi.1010939)

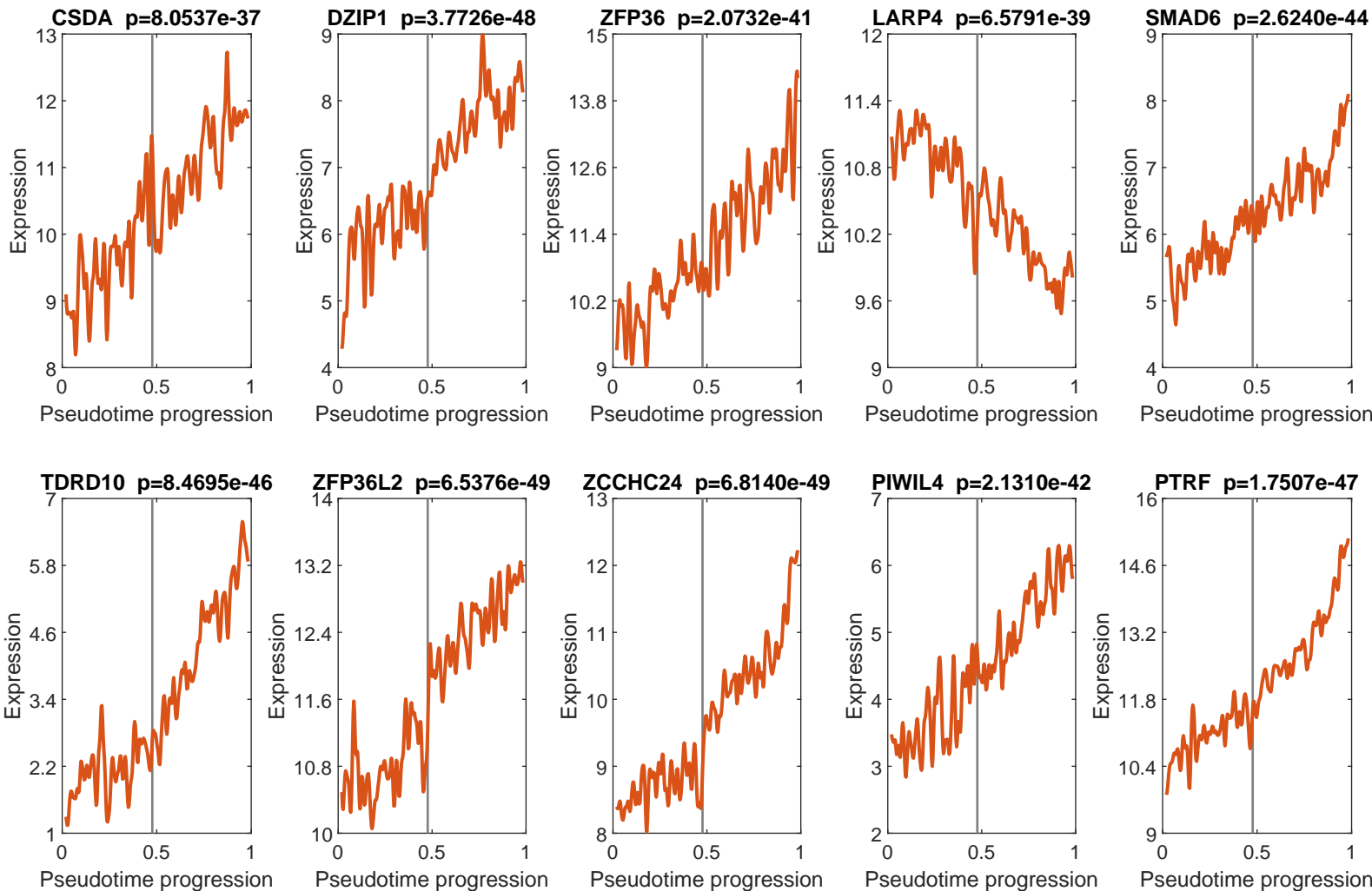

Supplement: S1 Fig — Most of these 10 RNA-binding proteins increased during the EM transition, and only LARP4 decreased. (PDF) [file pcbi.1010939.s001.pdf]

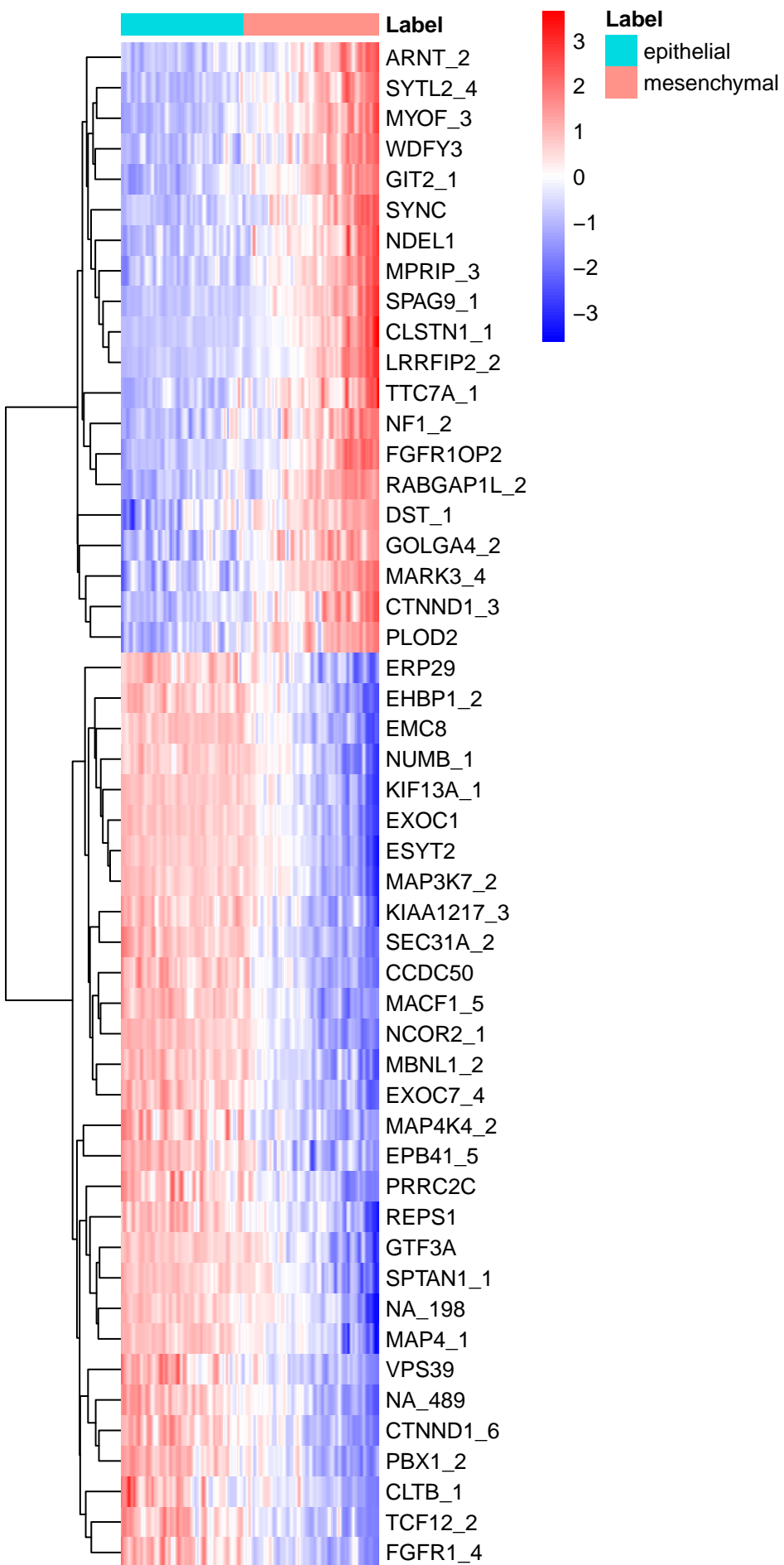

Supplement: S2 Fig — 50 alternative splicing events were clearly clustered into two groups: a descending group and an ascending group. (PDF) [file pcbi.1010939.s002.pdf]
