# Supplementary material for: PCB: A pseudotemporal causality-based Bayesian approach to identify EMT-associated regulatory relationships of AS events and RBPs during breast cancer progression
Source: PLoS Comput Biol. 2023 Mar 17;19(3):e1010939. doi: 10.1371/journal.pcbi.1010939 (PMC10057809; doi:10.1371/journal.pcbi.1010939)
Supplement: S3 Fig — The MCC value was calculated for each RNA-binding protein. The larger the MCC value, the darker the color in the figure. (A) RNA-binding protein network in the epithelial state. Here, ZCCHC24 was identified as the most influential RNA-binding protein. (B) RNA-binding protein network in the mesenchymal state. Here, PIWIL4 was identified as the most influential RNA-binding protein. (PDF) [file pcbi.1010939.s003.pdf]

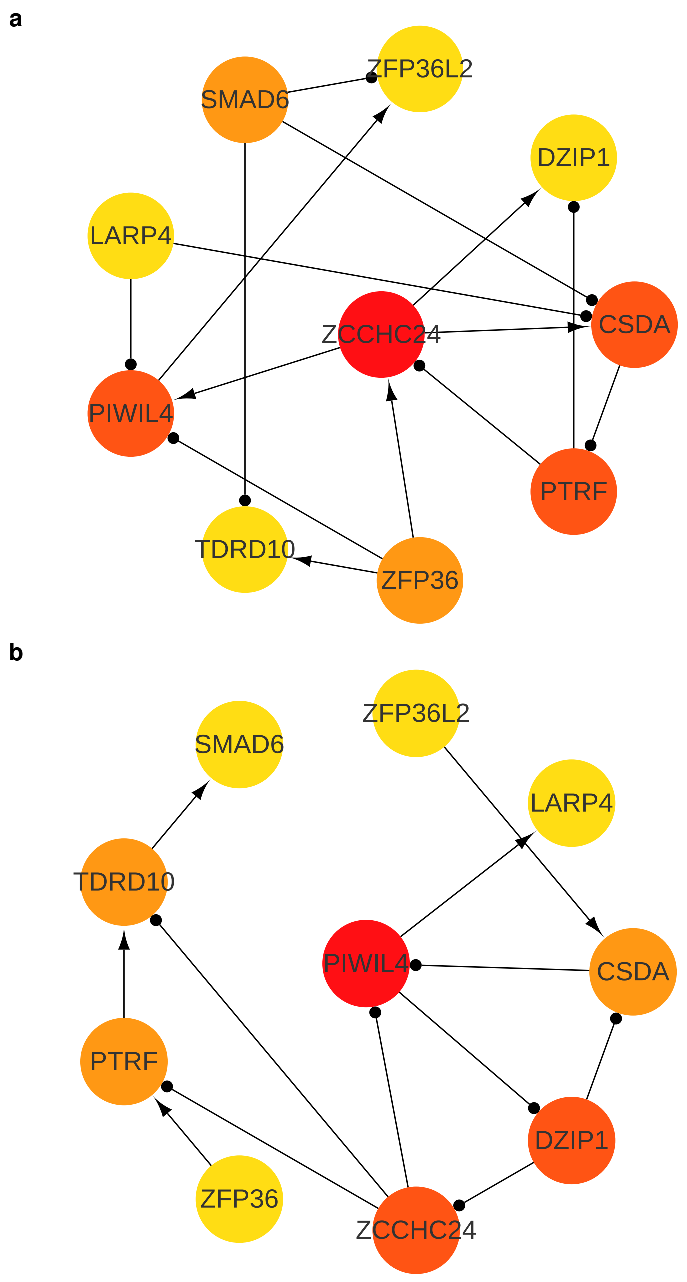

The mutual regulatory relationships between 10 RBPs. The MCC value was calculated for each RBP. The larger the MCC value, the darker the color in the figure. (a) RBP-RBP network in the epithelial state. Here, ZCCHC24 was identified as the most influential RBP. (b) RBP-RBP network in the mesenchymal state. Here, PIWIL4 was identified as the most influential RBP.
