# Supplementary material for: PCB: A pseudotemporal causality-based Bayesian approach to identify EMT-associated regulatory relationships of AS events and RBPs during breast cancer progression
Source: PLoS Comput Biol. 2023 Mar 17;19(3):e1010939. doi: 10.1371/journal.pcbi.1010939 (PMC10057809; doi:10.1371/journal.pcbi.1010939)
Supplement: S1 Text — (PDF) [file pcbi.1010939.s012.pdf]

PSEUDOTIME ANALYSIS

We applied a cross-sectional data of breast cancer patients with 143 epithelial specimens and 157 mesenchymal specimens out of a total of 1215 TCGA-BRCA samples [1], and used the pseudotime analysis to transform static dataset into a time series dataset. Specifically, the temporal progression inference was performed to quantitatively order samples based on the whole RNA-binding protein expression profile with epithelial specimens and mesenchymal specimens labeled by 1 and 2, respectively. The algorithm for pseudotime analysis [2] is described as follows:

1) Stage-weighted and locally scaled Gaussian kernel:

$$S(x, y) = \exp(-\gamma \|T_x - T_y\|^2), \tag{1}$$

where the parameter

$$\gamma = \frac{\omega_{xy}}{\varepsilon_x^2 + \varepsilon_y^2},$$

ω_{xy} is a weight coefficient given by EM transition process, which is defined in this study as $\omega_{xy} = 1 + |G_x - G_y|$, with the grades G_x and G_y representing EM transition process information of two specimens x and y, respectively. If a specimen x belongs to epithelial specimens, then $G_x = 1$; If a specimen x belongs to mesenchymal specimen, then $G_x = 2$. Moreover, the parameter ε_x is adaptive for each specimen x and is set as the specimen's distance to κ-th nearest neighbor. More specifically, we calculate the Euclidean distance between the RNA-binding protein profile of specimen x and the RNA-binding protein profile of the other sample. For specimen x, we can get a total of 300 Euclidean distances, sort these Euclidean distances, and assign the third-smallest Euclidean distance value (here let $\kappa = 3$) to ε_x. For example, consider the following dataset,

**Specimen**	**RBP1**	**RBP2**	**RBP3**
TCGA-BH-A0C7-01	11.7	8.9	9.6
TCGA-A8-A06N-01	12.3	9.0	9.7
TCGA-D8-A1XV-01	11.9	8.6	9.3
TCGA-E9-A1N3-01	11.9	8.7	9.1

For the specimen TCGA-BH-A0C7-01, the Euclidean distances are shown as below

**Euclidean distance**	TCGA-BH-A0C7-01
TCGA-BH-A0C7-01	0
TCGA-A8-A06N-01	0.6164
TCGA-D8-A1XV-01	0.4690
TCGA-E9-A1N3-01	0.5745

If $\kappa = 3$, then specimen TCGA-BH-A0C7-01's distance to 3-th nearest neighbor is 0.5745. T_x and T_y are vectors used to represent the RNA-binding protein expression profiles of the respective specimens x and y, $\|T_x - T_y\|$ is the L^2 norm of $T_x - T_y$.

2) Normalization of S:

$$H_{xy} = \frac{S(x,y)}{D(x)D(y)}, \tag{2}$$

where $D(x) = \sum_{y \in \Omega} S(x,y)$ and Ω is the set of all specimens.

3) Then a transition probability matrix $P = (P_{xy})$ is defined, where

$$P_{xy} = E(x)^{-\frac{1}{2}} H_{xy} E(y)^{-\frac{1}{2}}, \tag{3}$$

and $E(x) = \sum_{y \in \Omega} H_{xy}$ is the row normalization of H.

4) The accumulated transition probability:

$$Q = [I - (P - \psi_0 \psi_0^{\mathrm{T}})]^{\dagger} - I, \tag{4}$$

where ψ_0 is the first eigenvector of P (corresponding to eigenvalue 1) and $(I-(P-\psi_0\psi_0^{\mathrm{T}}))^{\dagger}$ is the generalized inverse (or Moore-Penrose inverse) of $I - (P - \psi_0 \psi_0^{\mathrm{T}})$.

5) A pseudotime distance between two specimens is defined as follows:

$$PD(x,y) = \|Q(x,\cdot) - Q(y,\cdot)\|, \tag{5}$$

where $\|\cdot\|$ is the L^2 norm.

6) The root sample x_0 can be identified according to the following formula:

$$x_0 = \arg \max_{x \in \{x_{\min}\}} PD(x, x_{ref}), \tag{6}$$

where x_{ref} is a randomly selected specimen from the maximal grade subpopulation, i.e., mesenchymal specimen subpopulation. The selection of x_0 was limited among specimens with the smallest garde subpopulation $\{x_{\min}\}$, i.e., epithelial specimen subpopulation, to eliminate potential influence of a few outliers in the data.

7) Finally, we can obtain the pseudotime score for each specimen x as follows:

$$s = PD(x, x_0). \tag{7}$$

According to the pseudotime score, the corresponding specimens are arranged in ascending order, and the sorted samples are mapped to a smoothed temporal trajectory.

REFERENCES

[1] Qiu,Y. *et al*. (2020). A combinatorially regulated RNA splicing signature predicts breast cancer EMT states and patient survival, *RNA*, **26(9)**, 1257–1267.

[2] Sun,X. *et al*. (2021). Inferring latent temporal progression and regulatory networks from cross-sectional transcriptomic data of cancer samples, *PLoS Computational Biology*, **17(3)**,e1008379.
