# Supplementary material for: PCB: A pseudotemporal causality-based Bayesian approach to identify EMT-associated regulatory relationships of AS events and RBPs during breast cancer progression
Source: PLoS Comput Biol. 2023 Mar 17;19(3):e1010939. doi: 10.1371/journal.pcbi.1010939 (PMC10057809; doi:10.1371/journal.pcbi.1010939)
Supplement: S2 Text — (PDF) [file pcbi.1010939.s013.pdf]

## TREND ANALYSIS

The trend analysis method is as follows:

**Algorithm 1: The trend analysis method**

1: Input: smoothed expression data $X_i$.
2: Fit $X_i$ to a linear function $L_i \times s + C_i$. Here, the estimated coefficient is denoted as $L_i$ and the constant is denoted as $C_i$.
3: Calculate the standard deviation of the detrended expression data, and denote as $V_i$. The detrended expression data $H_i(s)$ is obtained as $H_i(s) = X_i(s) - (L_i \times s + C_i)$, which means that removes the best straight-line fit linear trend from $X_i$. Therefore, $V_i = std(H_i(s))$.
4: A score is defined, $R_i = |L_i/V_i|$.
5: Output: score $R_i$.

Linear trend represents a systematic increase or decrease in data over time. By removing the linear trend from the data, the fluctuation of the detrended data was considered, and such fluctuation is not caused by time change. Therefore, we believe that the higher the score $R_i$, the most likely the expression of the corresponding alternative splicing event $i$ (RNA-binding protein $i$) changes significantly in the EM transition process.

An example is given to further illustrate. Consider the following data,

| $s$ | 0.0200 | 0.0233 | 0.0267 | 0.0300 | 0.0333 | ... | 0.9767 | 0.9800 | 0.9833 |
|---|---|---|---|---|---|---|---|---|---|
| $X_1$ | 3.1424 | 3.1370 | 3.1372 | 3.1414 | 3.1523 | ... | 3.0517 | 3.1227 | 3.1827 |

Fit $X_i$ to a linear function $L_i \times s + C_i$. Here, we get $L_1 = 0.5195$ and $C_1 = 2.9909$, i.e., the linear trend is $0.5195 \times s + 2.9909$. Then the detrended expression data $H_1(s) = X_i(s) - (0.5195 \times s + 2.9909)$. We have the score $R_1 = 3.5771$. The figure is as follows,

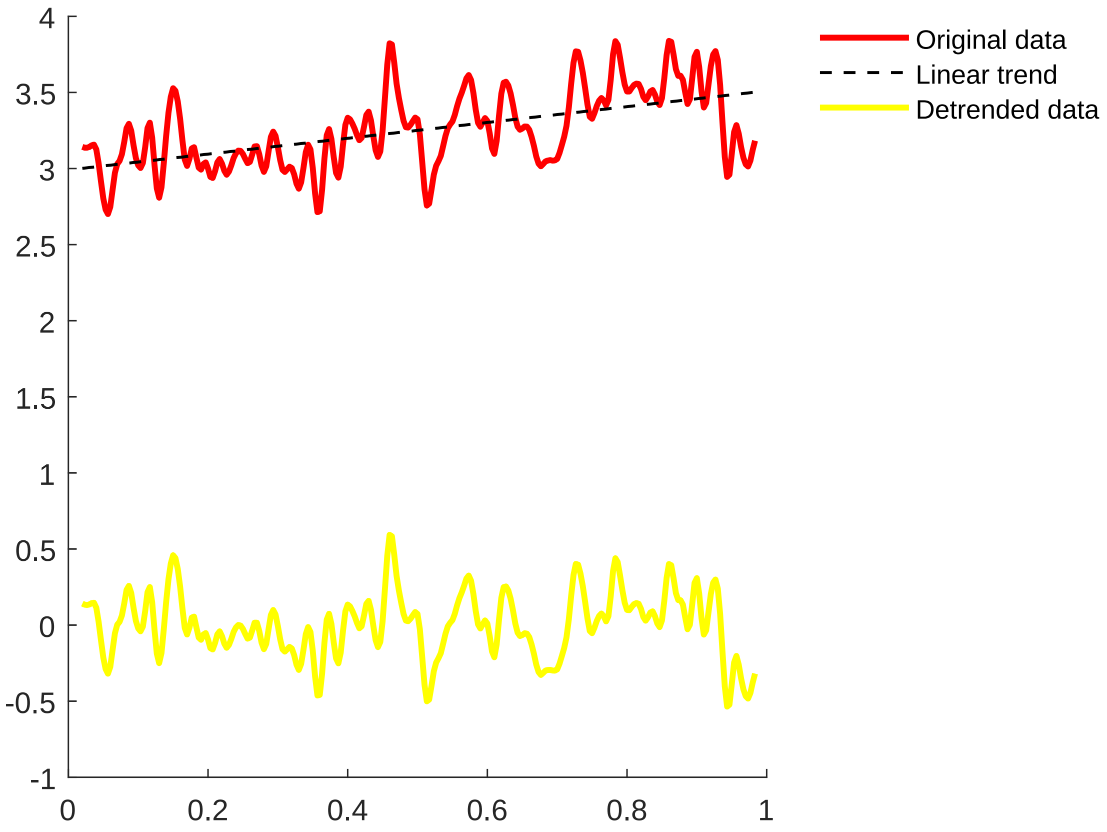
Original data
Linear trend
Detrended data
4
3.5
3
2.5
2
1.5
1
0.5
0
-0.5
-1
0
0.2
0.4
0.6
0.8
1
