# Supplementary material for: PCB: A pseudotemporal causality-based Bayesian approach to identify EMT-associated regulatory relationships of AS events and RBPs during breast cancer progression
Source: PLoS Comput Biol. 2023 Mar 17;19(3):e1010939. doi: 10.1371/journal.pcbi.1010939 (PMC10057809; doi:10.1371/journal.pcbi.1010939)
Supplement: S3 Text — (PDF) [file pcbi.1010939.s014.pdf]

MODEL OF RBP-AS EVENT REGULATORY RELATIONSHIPS

As described in [1], sequence-specific RNA-binding proteins bind to pre-mRNA to control alternative splicing. And each alternative splicing event is controlled by multiple RNA-binding proteins. Hence, alternative splicing events are dependent on RNA-binding proteins, but not the other way around. Based on the mass action kinetics [2], [3], [4] and the above priori knowledge, the regulatory relationships between alternative splicing events and RNA-binding proteins can be described by means of the following dynamical system,

$$\frac{\mathrm{d}X_i(s)}{\mathrm{d}s} = \sum_{j\neq i} a_{ij}X_i(s)\cdot X_j(s) + \sum_{l=1}^{M} b_{il}X_i(s)\cdot U_l(s) - d_iX_i(s), \quad i = 1, 2, \ldots, N, \tag{1}$$

$$\frac{\mathrm{d}U_l(s)}{\mathrm{d}s} = \sum_{k\neq l} c_{lk}U_l(s)\cdot U_k(s) - d'_lU_l(s), \quad l = 1, 2, \ldots, M, \tag{2}$$

where $X_i(s)$ and $U_l(s)$ represent the expression level of alternative splicing event $i,\ i = 1, 2, \ldots, N$ and RNA-binding protein $l,\ l = 1, 2, \ldots, M$ in breast cancer with pseudotime progression status s, respectively. Moreover, a_{ij} is the dynamic regulatory coefficient from alternative splicing event j to alternative splicing event i, where $i \neq j$ and $i, j = 1, 2, \ldots, N$; b_{il} is the dynamic regulatory coefficient from RNA-binding protein l to alternative splicing event i, where $i = 1, 2, \ldots, N$ and $l = 1, 2, \ldots, M$; c_{lk} is the dynamic regulatory coefficient from RNA-binding protein k to RNA-binding protein l, where $l \neq k$ and $l, k = 1, 2, \ldots, M$, and d_i is the self-degradation rate of alternative splicing event i; d'_l is the self-degradation rate of RNA-binding protein l.

The details of model assumption and derivative are provided as follows.

Let $X_i(t, s)$ and $U_l(t, s)$ represent the expression level of alternative splicing event $i,\ i = 1, 2, \ldots, N$ and RNA-binding protein $l,\ l = 1, 2, \ldots, M$ at time t in breast cancer with pseudotime progression status s, respectively. Assume cancer progression is an irreversible process over time so that $s = \varphi(t)$ is a strictly monotonic increasing function of t, i.e., $\varphi'(t) = \delta > 0$. As such, $s = \varphi(t)$ has an inverse function $t = \varphi^{-1}(s)$ and $\frac{\mathrm{d}t}{\mathrm{d}s} = \frac{1}{\varphi'(t)} = \frac{1}{\delta}$.

The change rates of $X_i(t, s)$ and $U_l(t, s)$ after a small progression period Δs can be modeled

by the following two difference equations:

$$\frac{X_i(\varphi^{-1}(s+\Delta s), s+\Delta s) - X_i(t,s)}{\Delta s} \\ = \sum_{j\neq i} a_{ij} X_i(t,s)\cdot X_j(t,s) + \sum_{l=1}^{M} b_{il} X_i(t,s)\cdot U_l(t,s) - d_i X_i(t,s), \tag{3}$$

$$\frac{U_l(\varphi^{-1}(s+\Delta s), s+\Delta s) - U_l(t,s)}{\Delta s} = \sum_{k\neq l} c_{lk} U_l(t,s)\cdot U_k(t,s) - d'_l U_l(t,s), \tag{4}$$

where a_{ij} is the dynamic regulatory coefficient from alternative splicing event j to alternative splicing event $i,\ i \neq j$; b_{il} is the dynamic regulatory coefficient from RNA-binding protein l to alternative splicing event i; c_{lk} is the dynamic regulatory coefficient from RNA-binding protein k to RNA-binding protein $l,\ l \neq k$; d_i is the self-degradation rate of alternative splicing event i; and d'_l is the self-degradation rate of RNA-binding protein l.

As $\Delta s \to 0$, we obtain the following progression-structured model in the form of partial differential equations (PDEs),

$$\frac{\partial X_i}{\partial t}\cdot(\varphi^{-1}(s))' + \frac{\partial X_i}{\partial s} = \sum_{j\neq i} a_{ij} X_i(t,s)\cdot X_j(t,s) + \sum_{l=1}^{M} b_{il} X_i(t,s)\cdot U_l(t,s) - d_i X_i(t,s), \tag{5}$$

$$\frac{\partial U_l}{\partial t}\cdot(\varphi^{-1}(s))' + \frac{\partial U_l}{\partial s} = \sum_{k\neq l} c_{lk} U_l(t,s)\cdot U_k(t,s) - d'_l U_l(t,s). \tag{6}$$

Since gene regulations or biochemical reactions are notably faster than cancer progression, we could assume that, in the above equations, $X_i(t,s)$ and $U_l(t,s)$ quickly approach its steady-state $\bar{X}_i(s)$ and $\bar{U}_l(s)$ as s changes, respectively, that is, $\frac{\partial \bar{X}_i(s)}{\partial t} = 0$ and $\frac{\partial \bar{U}_l(s)}{\partial t} = 0$. Therefore, we have the following ordinary differential equations (ODEs):

$$\frac{\mathrm{d}\bar{X}_i(s)}{\mathrm{d}s} = \frac{\partial \bar{X}_i(s)}{\partial s} = \sum_{j\neq i} a_{ij} \bar{X}_i(s)\cdot \bar{X}_j(s) + \sum_{l=1}^{M} b_{il} \bar{X}_i(s)\cdot \bar{U}_l(s) - d_i \bar{X}_i(s), \tag{7}$$

$$\frac{\mathrm{d}\bar{U}_l(s)}{\mathrm{d}s} = \frac{\partial \bar{U}_l(s)}{\partial s} = \sum_{k\neq l} c_{lk} \bar{U}_l(s)\cdot \bar{U}_k(s) - d'_l \bar{U}_l(s). \tag{8}$$

For simplicity, when there is no ambiguity, we write $\bar{X}_i(s)$ as $X_i(s)$ and $\bar{U}_l(s)$ as $U_l(s)$. (7) and (8) can therefore be rewritten as

$$\frac{\mathrm{d}X_i(s)}{\mathrm{d}s} = \sum_{j\neq i} a_{ij} X_i(s)\cdot X_j(s) + \sum_{l=1}^{M} b_{il} X_i(s)\cdot U_l(s) - d_i X_i(s), \tag{9}$$

$$\frac{\mathrm{d}U_l(s)}{\mathrm{d}s} = \sum_{k\neq l} c_{lk} U_l(s)\cdot U_k(s) - d_l' U_l(s). \tag{10}$$

Take $m+1$ points $s_i = s(r_i)$ from the smoothed progression trajectory $s(r)$, where $r_i = \frac{i}{m},\ i = 0,1,\dots,m$. We approximate

$$\frac{\mathrm{d}X_i(s_t)}{\mathrm{d}s} \approx \frac{X_i(s_{t+1}) - X_i(s_t)}{s_{t+1}-s_t}, \quad \frac{\mathrm{d}U_l(s_t)}{\mathrm{d}s} \approx \frac{U_l(s_{t+1}) - U_l(s_t)}{s_{t+1}-s_t}, \tag{11}$$

and denote

$$Y_{it} = \frac{X_i(s_{t+1}) - X_i(s_t)}{s_{t+1}-s_t}, \quad Z_{lt} = \frac{U_l(s_{t+1}) - U_l(s_t)}{s_{t+1}-s_t}, \tag{12}$$

where $s_{t+1}-s_t$ is sufficiently small (since m could be chosen large enough). Therefore, the above continuous model (i.e., (1) and (2)) can be discretized and rewritten as

$$Y_{it} \approx \sum_{j\neq i} a_{ij} X_i(s_t)\cdot X_j(s_t) + \sum_{l=1}^{M} b_{il} X_i(s_t)\cdot U_l(s_t) - d_i X_i(s_t), \tag{13}$$

$$Z_{lt} \approx \sum_{k\neq l} c_{lk} U_l(s_t)\cdot U_k(s_t) - d_l' U_l(s_t), \tag{14}$$

where $t = 0,1,\dots,m-1$.

We then denote

$$Y_i = (Y_{i0},\dots,Y_{it},\dots,Y_{i,m-1})^{\mathrm{T}}, \tag{15}$$

$$Z_l = (Z_{l0},\dots,Z_{lt},\dots,Z_{l,m-1})^{\mathrm{T}}, \tag{16}$$

$$A_i = (a_{i1},a_{i2},\dots,a_{iN},b_{i1},b_{i2},\dots,b_{iM},-d_i)^{\mathrm{T}}, \quad a_{ii}=0, \tag{17}$$

$$C_l = (c_{l1},c_{l2},\dots,c_{lM},-d_l')^{\mathrm{T}}, \quad c_{ll}=0, \tag{18}$$

and

$$X^{(i)} = \begin{bmatrix} X_i(s_0)X_1(s_0) & X_i(s_1)X_1(s_1) & \cdots & X_i(s_{m-1})X_1(s_{m-1}) \\ X_i(s_0)X_2(s_0) & X_i(s_1)X_2(s_1) & \cdots & X_i(s_{m-1})X_2(s_{m-1}) \\ \cdots & \cdots & \cdots & \cdots \\ X_i(s_0)X_N(s_0) & X_i(s_1)X_N(s_1) & \cdots & X_i(s_{m-1})X_N(s_{m-1}) \\ X_i(s_0)U_1(s_0) & X_i(s_1)U_1(s_1) & \cdots & X_i(s_{m-1})U_1(s_{m-1}) \\ X_i(s_0)U_2(s_0) & X_i(s_1)U_2(s_1) & \cdots & X_i(s_{m-1})U_2(s_{m-1}) \\ \cdots & \cdots & \cdots & \cdots \\ X_i(s_0)U_M(s_0) & X_i(s_1)U_M(s_1) & \cdots & X_i(s_{m-1})U_M(s_{m-1}) \\ X_i(s_0) & X_i(s_1) & \cdots & X_i(s_{m-1}) \end{bmatrix}^{\mathrm{T}}, \tag{19}$$

$$U^{(l)} = \begin{bmatrix} U_l(s_0)U_1(s_0) & U_l(s_1)U_1(s_1) & \cdots & U_l(s_{m-1})U_1(s_{m-1}) \\ U_l(s_0)U_2(s_0) & U_l(s_1)U_2(s_1) & \cdots & U_l(s_{m-1})U_2(s_{m-1}) \\ \cdots & \cdots & \cdots & \cdots \\ U_l(s_0)U_M(s_0) & U_l(s_1)U_M(s_1) & \cdots & U_l(s_{m-1})U_M(s_{m-1}) \\ U_l(s_0) & U_l(s_1) & \cdots & U_l(s_{m-1}) \end{bmatrix}^{\mathrm{T}}. \tag{20}$$

Consequently, (13) and (14) can be transformed into the following linear regression model:

$$Y_i = X^{(i)}A_i + \varepsilon_i, \quad i = 1, 2, \ldots, N, \tag{21}$$

$$Z_l = U^{(l)}C_l + \varepsilon_l', \quad l = 1, 2, \ldots, M, \tag{22}$$

where $\varepsilon_i = (\varepsilon_{i0}, \varepsilon_{i1}, \ldots, \varepsilon_{i,m-1})^{\mathrm{T}}$ and $\varepsilon_l' = (\varepsilon_{l0}', \varepsilon_{l1}', \ldots, \varepsilon_{l,m-1}')^{\mathrm{T}}$ are the random effects. Here, each $\varepsilon_{ik},\ k = 0, 1, \ldots, m-1$ is the random disturbance a mean of zero and $Cov(\varepsilon_i) = \sigma_i^2 I_m$ and similarly, each $\varepsilon_{lk}',\ k = 0, 1, \ldots, m-1$ is the random disturbance with a mean of zero and $Cov(\varepsilon_l') = {\sigma'}_l^2 I_m$.

Now, we use an adapted Bayesian Lasso method to infer the posterior distribution over the coefficients in each A_i. The method of inferring the posterior distribution over the coefficients in each C_l is similar, so we will only describe in detail the process of inferring the posterior distribution over the coefficients in each A_i. First, the above assumptions imply that the data likelihood is

$$\ell(A_i, \sigma_i^2 | Y_i, X^{(i)}) = \prod_{t=0}^{m-1} \phi(Y_{it}; X_t^{(i)} A_i, \sigma_i^2), \tag{23}$$

where $t = 0, 1, \ldots, m-1$, $X_t^{(i)}$ is the $t+1$-th row of $X^{(i)}$ and $\phi(Y_{it}; X_t^{(i)} A_i, \sigma_i^2)$ is the Gaussian probability density with mean $X_t^{(i)} A_i$ and variance σ_i^2 evaluated at Y_{it}.

Then we assume that these prior distributions:

- $A_i | \sigma_i^2, \lambda_i$ has a Laplace distribution with a mean of 0 and a scale of $\frac{\sigma_i^2}{\lambda_i}$, where λ_i is the shrinkage parameter, which is set to 1. The coefficients are conditionally independent.
- $\sigma_i^2 \sim IG(A, B)$, where A and B are the shape and scale, respectively, of an inverse gamma distribution.

Using Bayes' rule, we formulate the joint posterior distribution of A_i and σ_i^2 as follows:

$$\pi(A_i, \sigma_i^2 | Y_i, X^{(i)}) \propto \pi(A_i | \sigma_i^2, \lambda_i) \cdot \pi(\sigma_i^2) \cdot \ell(A_i, \sigma_i^2 | Y_i, X^{(i)}). \tag{24}$$

Perform Bayesian lasso regression by passing the prior model and data to estimate, that is, by estimating the posterior distribution of A_i and σ_i^2 (C_l and σ'^2_l). Then we use Markov chain Monte Carlo (MCMC) algorithm to sample from the posterior. A directed edge from alternative splicing event j (RNA-binding protein l) to alternative splicing event i could be determined to be presented if the 95% credible interval (CI) of the parameter estimates of a_{ij} (b_{il}) does not contain zero, otherwise absent. Similarly, a directed edge from RNA-binding protein k to RNA-binding protein l could be determined to be presented if the 95% credible interval (CI) of the parameter estimates of c_{lk} does not contain zero, otherwise absent.

REFERENCES

[1] Fu XD. *et al*. (2014). Context-dependent control of alternative splicing by RNA-binding proteins, *Nature Reviews Genetics*, **15(10)**, 689–701.

[2] Alves F. *et al*. (2021). A simple framework to describe the regulation of gene expression in prokaryotes, *Comptes Rendus Biologies*, **328(5)**, 429–444.

[3] Chan TE. *et al*. (2017). Gene regulatory network inference from single-cell data using multivariate information measures, *Cell Systems*, **5(3)**, 251–267.

[4] Sun X. *et al*. (2021). Inferring latent temporal progression and regulatory networks from cross-sectional transcriptomic data of cancer samples, *PLoS Computational Biology*, **17(3)**, e1008379.
