# Supplementary material for: PCB: A pseudotemporal causality-based Bayesian approach to identify EMT-associated regulatory relationships of AS events and RBPs during breast cancer progression
Source: PLoS Comput Biol. 2023 Mar 17;19(3):e1010939. doi: 10.1371/journal.pcbi.1010939 (PMC10057809; doi:10.1371/journal.pcbi.1010939)
Supplement: S4 Text — (PDF) [file pcbi.1010939.s015.pdf]

TESTING THE MODEL WITH A SYNTHETIC DATASET

In order to evaluate the effectiveness and accuracy of our proposed model RNA-binding protein, we generated a set of synthetic cross-sectional expression data. For visualization, we considered 3 RNA-binding proteins and 5 alternative splicing events in 100 cancer specimens.

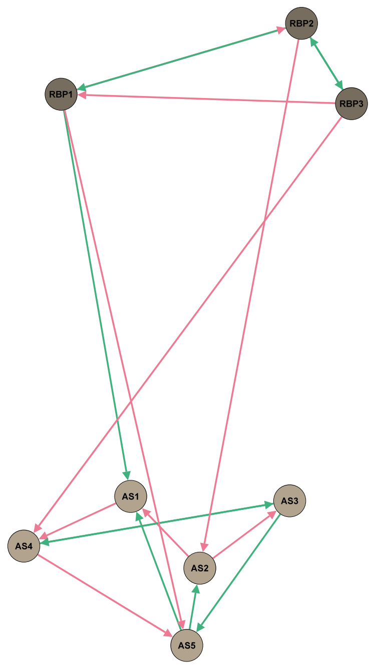

Under the same assumptions of dynamical systems in the main text, the following ordinary differential equations (ODEs) were constructed to produce changes in the expression of RNA-binding proteins and alternative splicing events with the EM transition process of cancer,

$$\frac{\mathrm{d}X_i(s)}{\mathrm{d}s} = \sum_{j\neq i} a_{ij}X_i(s)\cdot X_j(s) + \sum_{l=1}^{3} b_{il}X_i(s)\cdot U_l(s) - d_iX_i(s), \quad i = 1,2,\ldots,5, \tag{1}$$

$$\frac{\mathrm{d}U_l(s)}{\mathrm{d}s} = \sum_{k\neq l} c_{lk}U_l(s)\cdot U_k(s) - d_l'U_l(s), \quad l = 1,2,\ldots,3, \tag{2}$$

where

$$(a_{ij})_{5\times5} = \begin{bmatrix} 0 & 2 & 0 & 0 & -2 \\ 0 & 0 & 0 & 0 & -1 \\ 0 & 1 & 0 & -1 & 0 \\ 4 & 0 & -3 & 0 & 0 \\ 0 & 0 & -2 & 1 & 0 \end{bmatrix}, (b_{il})_{5\times3} = \begin{bmatrix} -1 & 0 & 0 \\ 0 & 1 & 0 \\ 0 & 0 & 0 \\ 0 & 0 & 1 \\ 2 & 0 & 0 \end{bmatrix}, (c_{lk})_{3\times3} = \begin{bmatrix} 0 & -2 & 2 \\ 1 & 0 & -2 \\ 0 & -1 & 0 \end{bmatrix},$$

and degradation rates (d_i) and (d_l') were set as $(-1, -0.5, -1, -1, -0.5)^{\mathrm{T}}$ and $(-2, -2, -0.5)^{\mathrm{T}}$, respectively. The initial values of the expression level of each RNA-binding protein and the expression level of each alternative splicing event in the above ODEs were set to standardized 1. By numerically solving the above ODEs, we get two sets of time series data. We uniformly sampled data to simulate the RNA-binding protein expression profile and alternative splicing event expression profile of 100 patients ordered along with the EM transition process. Then, the sample IDs were randomly assigned, but the information that the data belongs to epithelial (mesenchymal) specimens was retained, that is, epithelial samples were all in S1 interval and mesenchymal samples were all in S2 interval.

Based on the sample-randomized of RNA-binding protein expression profile, we evaluated whether the proposed method could accurately order the samples. Based on the sorted sample data, we further evaluated whether the proposed method could effectively reconstruct the regulatory relationships between alternative splicing events and RNA-binding proteins.

Firstly, according to the given cross-sectional RNA-binding protein expression profile data, we used the developed graph-based random walk method to quantify the pseudotime distance from patients in the simulated cohort to the inferred "root". Comparing the pseudotime score of each patient with the true progression, it shows that the proposed model faithfully recovered the real order of samples (Spearman's rho=0.99946). Moreover, along with the pseudotime progression, both RNA-binding protein expression dynamics and alternative splicing event expression dynamics show very similar contours to the original data.

Then, we reconstructed the regulatory relationships between alternative splicing events and RNA-binding proteins by means of Bayesian Lasso method. For coefficient $[A_i]_{ij}$, where $A_i = (a_{i1}, a_{i2}, \ldots, a_{iN}, b_{i1}, b_{i2}, \ldots, b_{iM}, -d_i)^{\mathrm{T}}$ and $a_{ii} = 0$, if the $\alpha\%$ credible interval (CI) did not contain zero, there is interaction between alternative splicing event $j,\ j = 1, 2, \ldots, N$ (RNA-binding protein $j - N,\ j = N+1, N+2, \ldots, N+M$) and alternative splicing event i, otherwise there is no interaction between them. We defined the following score to quantify the presence probability of each predicted interaction from alternative splicing event $j,\ j = 1, 2, \ldots, N$ or RNA-binding protein $j - N,\ j = N+1, N+2, \ldots, N+M$ to alternative splicing event i,

$$S_{ij} = 1 - \inf\left(\alpha | 0 \notin CI_\alpha\left([A_i]_{ij}\right)\right), \tag{3}$$

where $CI_\alpha\left([A_i]_{ij}\right)$ is the $\alpha\%$ CI of posterior distribution of $[A_i]_{ij}$. For coefficient $[C_l]_{lk}$, where

$C_l = (c_{l1}, c_{l2}, \ldots, c_{lM}, -d_l')^{\mathrm{T}}$ and $c_{ll} = 0$, we also used the same method to judge whether there is interaction between any two RNA-binding proteins.

The area under curve (AUC) of receiving operator characteristic (ROC) with and without interaction was used as a metric to evaluate the effectiveness of our method in restoring the network structure. True positive rate (TPR) and false positive rate (FPR) for the inferred network compared to the ground-truth network (a_{ij}, b_{il}, c_{lk} in (1) and (2)) are defined by the following equations, respectively:

$$TPR = \frac{TP}{TP + FN}, \tag{4}$$

$$FPR = \frac{FP}{FP + TN}, \tag{5}$$

where TP, FP, TN and FN are the numbers of true positives, false positives, true negatives and false negatives, respectively. TPR and FPR were used to draw the ROC curves. We used the trapezoidal method for calculating the AUC of ROC.

Here, we demonstrated the accuracy of the model in terms of the above dynamical systems. The AUC of ROC could be calculated for the inferred network compared with the ground-truth network (a_{ij}, b_{il}, c_{lk} in (1) and (2)) based on the $\alpha\%$ CI that contained zero or not.
